# Nitrogen and carbon isotopic dynamics of subarctic soils and plants in southern Yukon Territory and its implications for paleoecological and paleodietary studies

**DOI:** 10.1371/journal.pone.0183016

**Published:** 2017-08-16

**Authors:** Farnoush Tahmasebi, Fred J. Longstaffe, Grant Zazula, Bruce Bennett

**Affiliations:** 1 Department of Earth Sciences, The University of Western Ontario, London, Ontario, Canada; 2 Yukon Palaeontology Program, Department of Tourism and Culture, Government of Yukon, Whitehorse, Yukon Territory, Canada; 3 Yukon Conservation Data Centre, Environment Yukon, Government of Yukon, Whitehorse, Yukon Territory, Canada; Odum School of Ecology, University of Georgia, UNITED STATES

## Abstract

We examine here the carbon and nitrogen isotopic compositions of bulk soils (8 topsoil and 7 subsoils, including two soil profiles) and five different plant parts of 79 C_3_ plants from two main functional groups: herbs and shrubs/subshrubs, from 18 different locations in grasslands of southern Yukon Territory, Canada (eastern shoreline of Kluane Lake and Whitehorse area). The Kluane Lake region in particular has been identified previously as an analogue for Late Pleistocene eastern Beringia. All topsoils have higher average total nitrogen *δ*^15^N and organic carbon *δ*^13^C than plants from the same sites with a positive shift occurring with depth in two soil profiles analyzed. All plants analyzed have an average whole plant *δ*^13^C of −27.5 ± 1.2 ‰ and foliar *δ*^13^C of –28.0 ± 1.3 ‰, and average whole plant *δ*^15^N of −0.3 ± 2.2 ‰ and foliar *δ*^15^N of –0.6 ± 2.7 ‰. Plants analyzed here showed relatively smaller variability in *δ*^13^C than *δ*^15^N. Their average *δ*^13^C after suitable corrections for the Suess effect should be suitable as baseline for interpreting diets of Late Pleistocene herbivores that lived in eastern Beringia. Water availability, nitrogen availability, spacial differences and intra-plant variability are important controls on *δ*^15^N of herbaceous plants in the study area. The wider range of *δ*^15^N, the more numerous factors that affect nitrogen isotopic composition and their likely differences in the past, however, limit use of the modern N isotopic baseline for vegetation in paleodietary models for such ecosystems. That said, the positive correlation between foliar *δ*^15^N and N content shown for the modern plants could support use of plant *δ*^15^N as an index for plant N content and therefore forage quality. The modern N isotopic baseline cannot be applied directly to the past, but it is prerequisite to future efforts to detect shifts in N cycling and forage quality since the Late Pleistocene through comparison with fossil plants from the same region.

## 1 Introduction and background information

Stable isotopes of carbon and nitrogen are valuable for studying food webs and tracking transfer of energy and materials through trophic levels [1]. Primary producers in different ecosystems provide varied food sources with distinct carbon (*δ*^13^C) and nitrogen (*δ*^15^N) isotopic compositions at the base of the food web. The *δ*^13^C and *δ*^15^N of an animal reflect both the isotopic composition of its diet and fractionations during the building of organic tissues. Hence, an isotopic baseline for each ecosystem should be established prior to comparing animals from different regions and times [1]. This is particularly important for paleoecological and paleodietary studies of Late Pleistocene subarctic ecosystems. Such investigations have a special value in the study of whole ecosystem (structure, composition and function) responses to climate change.

Stable isotope data have been widely used in the study of Late Pleistocene subarctic mammals e.g. [2–8]. Determinations of appropriate modern and past local and regional, foodweb isotopic baselines, however, are still underrepresented in the literature. Subarctic ecosystems are not homogeneous. Local and regional ecological mosaics likely existed in the past because of variation in topography, soil moisture, loess deposition, altitude and animal disturbance [9] and are present currently owing to the bioclimatic subzones, patterned ground created by soil-frost processes, different plant communities, and the changes in elevation [10]. The possibility of such microhabitat diversity should be considered when establishing isotopic baselines for these ecosystems.

Soils and plants, which can show an extremely wide range of *δ*^13^C and *δ*^15^N, are two main components of all terrestrial ecosystems [11, 12]. A number of studies have addressed plant *δ*^13^C [13, 14] and *δ*^15^N [13, 15–18] variations in Arctic and subarctic ecosystems in North America, Eurasia and Iceland. Wooller et al. [14] conducted the most comprehensive study, analyzing the *δ*^13^C of herbarium modern plants (around 200 taxa) as well as fossil plants from Alaska and Yukon Territory. Schulze et al. [17] studied N isotopic and elemental compositions of different plants in northern Alaska with the aim of investigating their difference in nutrient acquisition. Similar studies have reported *δ*^15^N for various plants from other parts of this ecosystem [15, 16]. The analysis of both *δ*^13^C and *δ*^15^N of plants has also been conducted for *Carex* from 15 Eurasian Coastal Arctic sites [18] and for lichens and plants from Iceland [13]. While these studies have added greatly to our general isotopic knowledge in these regions, additional local and regional isotopic studies are needed to evaluate the heterogeneity that can exist in these ecosystems.

**Fig 1:**
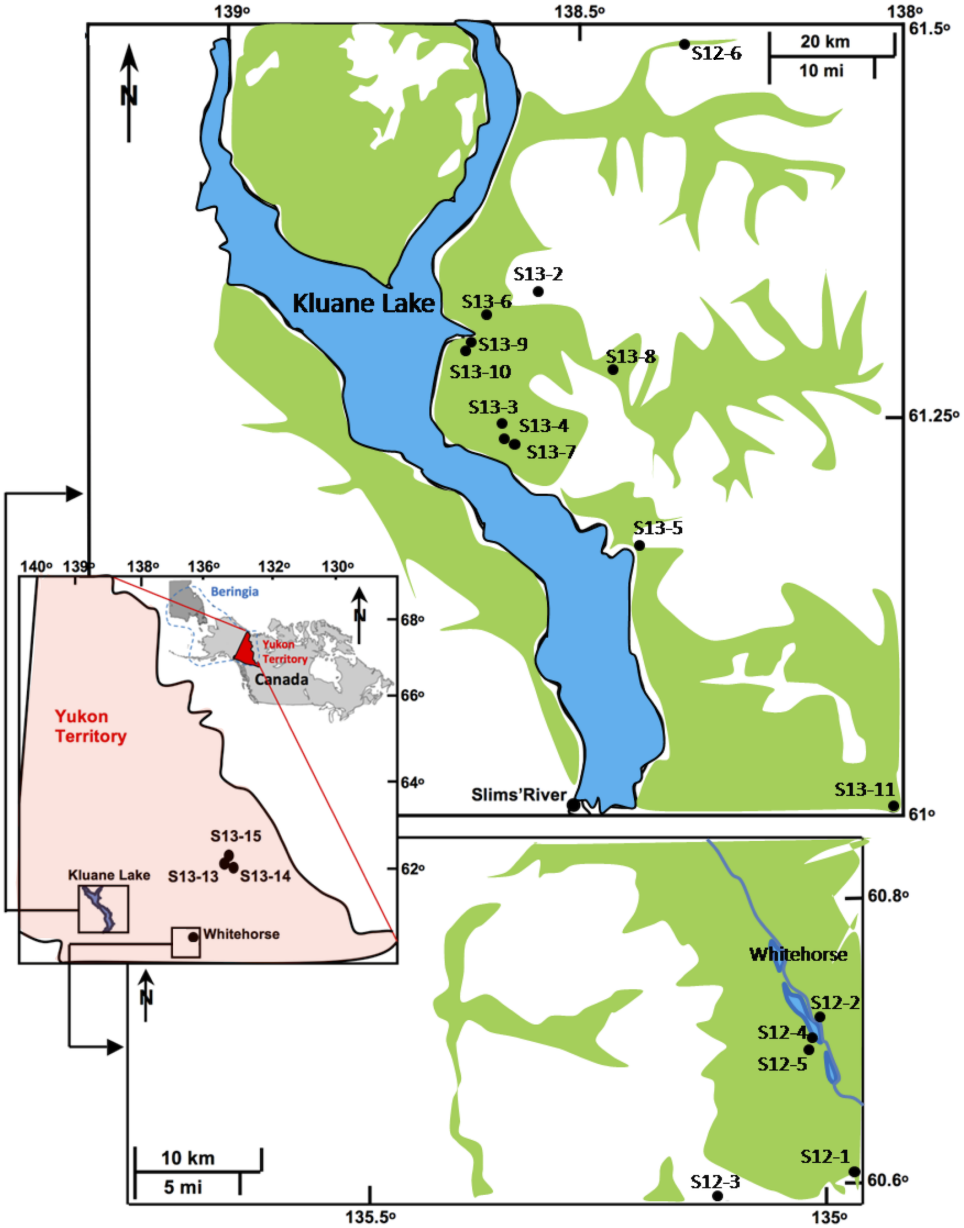
Location of study areas and sampling sites in Yukon Territory, Canada. The study area is located in the eastern part of Beringia (blue dash lines on the inset of Canada, Alaska and easternmost Asia).

In the present study, we measured the *δ*^13^C and *δ*^15^N of soils and plants from the south of Yukon Territory (eastern shoreline of Kluane Lake and areas around Whitehorse) (Fig 1). The study’s purpose is to establish a local isotopic baseline for modern foodwebs and to gain a better understanding of main environmental factors affecting C and N isotopic dynamics in this region. The results should also enable future work in which modern and Late Pleistocene isotopic baselines for eastern Beringia are compared, in order to identify environmental factors that affected the baseline. Beringia was a largely ice-free land extending from northwest Canada to northeast Siberia (Fig 1) [19] and was located within the Mammoth Steppe Ecosystem, which was the most extensive biome on Earth during the Last Glacial Maximum [20]. Previous reconstructions of Beringia [9, 19] have shown similarities in main ecosystem contexts (soil and grassland compositions) to the Kluane Lake area [21].

### 1.1 Study areas: Kluane Lake and Whitehorse, Yukon Territory

The two main regions investigated in this study are located in the southern Yukon Territory: (i) the eastern shoreline of Kluane Lake, and (ii) the Whitehorse area (Fig 1). The first site consists of grasslands located next to the southeast shore of Kluane Lake, which receives windblown loess from the Slims River delta. The second site consists of grasslands in the Whitehorse valley. Table 1 lists mean air temperature and total precipitation over the last 29 years at these sites as well as these data for 2012 and 2013, when the plant samples were collected. A few plant samples were also obtained from three sites (S13-13, -14, -15) in the Faro area (Fig 1).

**Table 1:** Climate data for Kluane Lake and Whitehorse study areas.

	29 year average (1981–2010)	2012 (WY)^a^	2013 (WY)	2012 (GSM)^b^	2013 (GSM)
**MAT (°C)**^c^					
KluaneLake^d^	-2.1	-3.0	-1.1	9.4	12.0
Whitehorse^e^	-0.1	-1.0	0.5	11.1	13.3
**MTP (mm)**^f^					
Kluane Lake	124.3	-	-	206.0	101.2
Whitehorse	262.3	275.0	266.6	170.7	95.9

^a^ **WY**: Whole Year.

^b^ **GSM**: Growing Season Months (May, June, July and August).

^c^ **MAT**: Mean Air Temperature (Data from Environment Canada, 2015).

^d^ Haines Junction station (60°45'9.7266" N, 137°30'37.5192" W).

^e^ Whitehorse station (60°43'59.000" N, 135°05'52.000" W).

^f^ **MTP**: Mean Total Precipitation (Data from Environment Canada, 2015).

Data prior to 1981 were not available to us.

The Kluane Ranges (2000–2800 masl), which are located in the southwest of Yukon Territory, effectively block penetration of Pacific air masses to the Kluane Plateau, resulting in a semiarid continental climate for the Kluane area with cold winters and warm summers [21]. The combination of ice fields at the core of the Kluane Ranges, from which glaciogenic silt and sand are delivered to the Slims River delta, strong winds off of these glaciers, and arid conditions, which amplify evapotranspiration, facilitate continuous transportation and accumulation of loess on the eastern side of Kluane Lake. Such conditions are similar to those reconstructed by Guthrie [22] for eastern Beringia loess formation during the Late Pleistocene.

**Fig 2:**
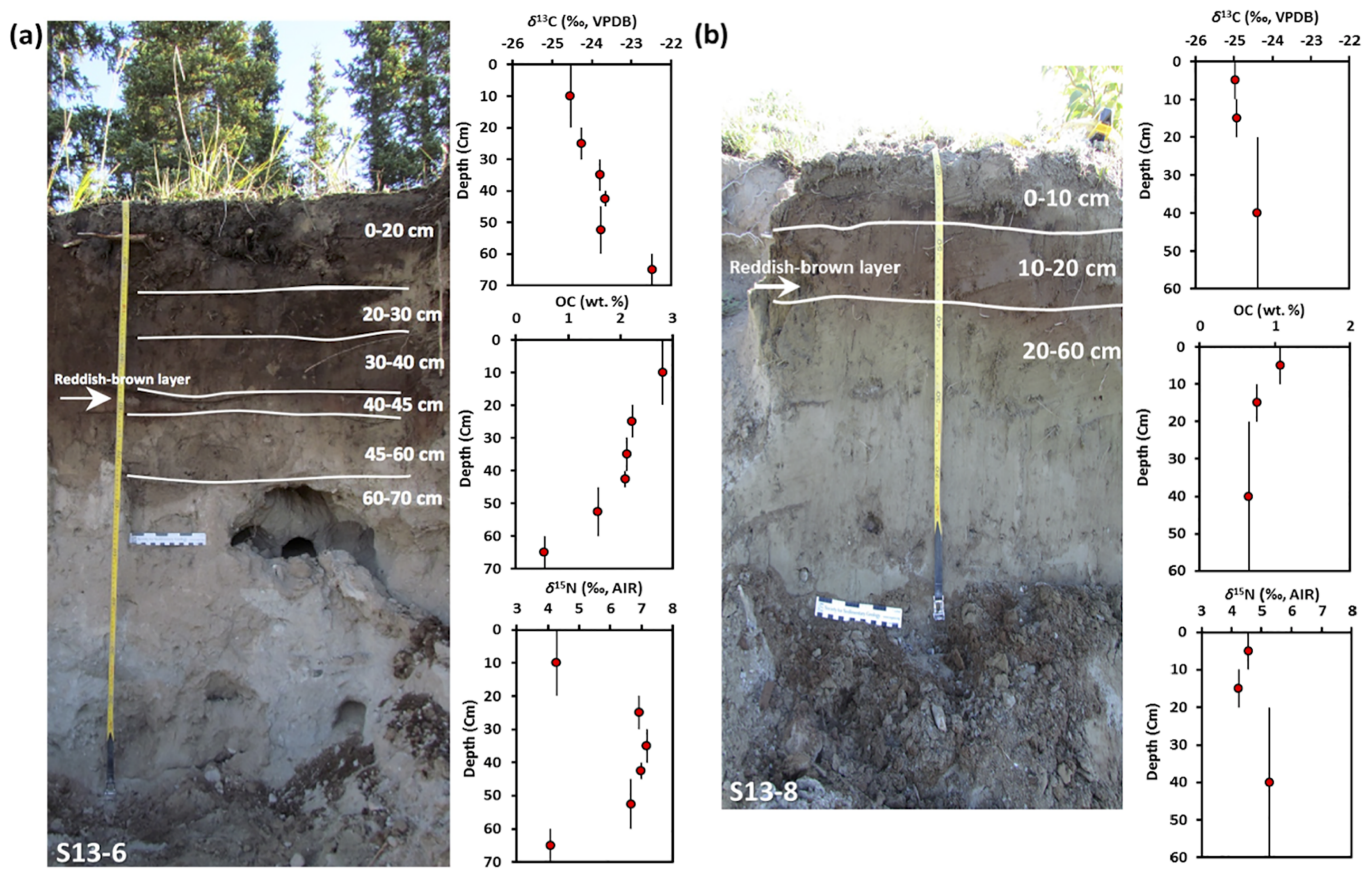
Soil profiles at Kluane Lake and their corresponding depth profiles for *δ*^13^C_OC_, OC and *δ*^15^N_TN_. (a) profile S13-6, and (b) profile S13-8. In both, data points provide average values for bulk analysis of each soil interval (indicated by the vertical lines).The reddish-brown layer is called the “Slims soil”, and separates Neoglacial loess deposits from underlying Late Pleistocene/early Holocene deposits (photographic credit: Tessa Plint).

Loess accumulation in this area occurred during two time periods: Late Pleistocene/early Holocene and Neoglacial [23]. The region still experiences frequent dust storms, particularly during summer months. In most soils from this area, the two loess phases are separated by a reddish-brown paleosol (Fig 2), which is named the “Slims soil” [24].

The vegetation of the Kluane region comprises a mixture of grassland and boreal forest ranging from valley-bottom elevations (781 m) to ~1160 m [25]. The grasslands are composed mainly of *Artemisia-Festuca* communities, which are predominant in drier parts of the area on southwest-facing aspects. The forests consist mainly of white spruce [21].

The Whitehorse valley, like most of Yukon Territory, has a dry, subarctic climate characterized by long and cold winters and short and cool summers. The long-term records (Table 1) show a west to east difference in mean total precipitation, with Whitehorse receiving significantly more precipitation than Kluane Lake. The location of the City of Whitehorse in this valley makes its climate milder than other areas of the Yukon (Table 1). The vegetation is more or less similar to the Kluane Lake area, consisting mainly of boreal forest and grasslands. Shrub communities are also present near the tree line, under a canopy of trees. Grasslands are limited to dry and south-facing slopes, while forests cover many plateaus and valleys [26].

### 1.2 Carbon isotopic composition of soil and terrestrial plants

#### 1.2.1 *δ*^13^C of soil organic carbon

Vegetation following different photosynthesis pathways (C_3_, C_4_ and CAM) imparts different *δ*^13^C signatures to soil organic carbon (SOC). This difference is the basis for many studies of past vegetation and climate change [27–30]. Other processes also affect the *δ*^13^C of SOC. A negative relationship has been observed between soil *δ*^13^C_OC_ and mean annual precipitation (MAP) in C_3_-dominated ecosystems [28]. This relationship is explained by plant C isotopic response to MAP, which is transmitted to soils [31]. Discrimination against ^13^C during microbial decomposition also affects soil *δ*^13^C_OC_. This effect depends mostly on the extent of decomposition, which is controlled by climate [32]. Increases in *δ*^13^C_OC_ with depth are typically attributed to microbial degradation when <4 ‰ [33, 34], and to C_3_/C_4_ vegetation change when >4 ‰ [35].

#### 1.2.2 Controls on plant *δ*^13^C

Among vascular plants, C_3_ plants are characterized by the lowest *δ*^13^C (–38 to –22 ‰), C_4_ plants have higher *δ*^13^C (–21 to –9 ‰) and CAM (Crassulacean Acid Metabolism) plants lie between (–30 to –13 ‰) [36–38]. C_3_ plants are overwhelmingly dominant in high latitudes [39, 14], including our study sites (above 60ᵒN).

Other factors affecting plant *δ*^13^C include the *δ*^13^C of source CO_2_, relative humidity/water availability, temperature, light intensity and partial pressure of CO_2_ (*p*CO_2_) [31, 36, 37, 40, 41]. Water availability is most important. A negative correlation between MAP and C_3_ plant *δ*^13^C [41–44] is attributed to stomatal closure in response to aridity, producing reduced C_i_/C_a_, higher water use efficiency and less negative *δ*^13^C [36–38]. Understory growth under closed tree canopies–the “canopy effect”–can lead to more negative plant *δ*^13^C due to fixing of ^13^C-depleted CO_2_ from soil and canopy respiration [45], lower light intensity and higher *p*CO_2_ [45]. No samples in the present study, however, grew under such conditions.

In C_3_ plants, photosynthesizing tissues (e.g. leaf) have more negative *δ*^13^C than heterotrophic tissues (e.g. stem, root, inflorescence) [46]. Causes include different macromolecular tissue compositions, growing stage variations in photosynthetic discrimination against ^13^C, and different contributions of day versus night sucrose with different *δ*^13^C to different tissues [46].

### 1.3 Nitrogen isotopic composition of soil and terrestrial plants

#### 1.3.1 *δ*^15^N of source N

Nitrate (NO_3_^-^), ammonium (NH_4_^+^) and dissolved organic compounds (e.g. simple proteins, amino acids and amino sugars) [47–49] are the most common forms of soil N taken up by plants. The *δ*^15^N varies among these sources because of different biochemical reactions in soil (N mineralization, nitrification, denitrification and volatilization) [50, 51]. Bulk soil *δ*^15^N, however, is not always a good representation of bioavailable N [50]. Adding to the complexity is the *δ*^15^N gradient with soil depth [49, 52]. Increasing bulk soil *δ*^15^N with soil depth [53– 55] has been attributed to fresh litter input to topsoil, coupled with accumulation of ^15^N-enriched, decomposing organic matter with depth [54]. Plants have diverse abilities to acquire N depending on their rooting depth and phenology [55], life form (trees, shrubs and herbs) [17], and preference for different forms of N [16, 56] at different times of year [57].

Mycorrhizal fungi associations also affect the *δ*^15^N of source N for plants. Plants are likely more reliant on mycorrhizal fungi for N acquisition under conditions of low N availability [11], which commonly is the case in Arctic and subarctic ecosystems [58]. Arbuscular mycorrhizal-, ectomycorrhizal- and ericoid mycorrhizal-associated plants are depleted of ^15^N by 2 ‰, 3.2 ‰ and 5.9 ‰, respectively, relative to non-mycorrhizal plants [11, 59–61].

Herbivory (grazing, trampling, excretory products, soil disturbance) also can change soil and plant *δ*^15^N. Herbivory affects N dynamics by changing N availability [62], altering the rate of soil N processes [62], and modifying litter quality and plant composition [63, 64]. No systematic pattern of N-isotope effects, however, has emerged from herbivory [65–71].

#### 1.3.2 Controls on plant *δ*^15^N

While nitrogen in plants mainly originates from soil, plant *δ*^15^N varies from total soil N [12]. This reflects the range of bioavailable versus non-bioavailable N compounds in bulk soil N, isotopic fractionation during N uptake by plants, and biological processes during N assimilation [12, 50].

**1.3.2.1 Nitrogen Isotopic Fractionation during N Uptake and Assimilation.** The size of nitrogen isotope fractionation (ε) during plant uptake of NO_3_^-^ and NH_4_^+^ is controlled by two factors: (i) external N concentration [72, 73], and (ii) efflux of ^15^N-enriched inorganic N and/or ^15^N-depleted organic N from roots after N uptake [72, 74]. Values of ε are small during plant uptake under low concentrations of NO_3_^-^ and/or NH_4_^+^ (~0.5 mol m^3-^); ε increases at higher concentrations [75, 76]. Discrimination against ^15^N during plant uptake is negligible under most natural conditions [72, 77]. Enzymatic reactions during N assimilation typically produce large fractionations; ε of +15 ‰ and +17 ‰, respectively, have been reported for nitrate reductase and glutamine synthetase, two main enzymes involved in N assimilation [72].

**1.3.2.2 Intra-plant Variation in *δ***^**15**^**N.** Intra-plant variation in *δ*^15^N can arise from: (i) variation in plant nitrogen sources as different organs form and expand, (ii) different patterns of N assimilation with either NO_3_^-^ or NH_4_^+^ as the primary N source, (iii) reallocation and transportation of N macromolecules between sink and source organs, and (iv) organ-specific efflux of N [72]. Leaves normally have higher *δ*^15^N than other organs, particularly roots [72, 78], although there are some exceptions [79]. When NO_3_^-^ is the sole N source, significant intra-plant variation occurs, with leaves having much higher *δ*^15^N than roots. This likely reflects different patterns of NO_3_^-^ *vs*. NH_4_^+^ assimilation. Whereas NH_4_^+^ is assimilated immediately after root uptake, some NO_3_^-^ is assimilated in roots while the remaining, ^15^N-enriched NO_3_^-^ is transported to shoots for N assimilation in leaves [72].

Enzymatic reactions involved in reallocation of N also can produce molecules with lower *δ*^15^N than the original source and thus cause intra-plant *δ*^15^N variation [72]. Likewise, loss of NH_3_ through plant leaves and efflux of organic N from roots can enrich these organs in ^15^N [50, 72].

**1.3.2.3 Environmental Factors and Plant *δ***^**15**^**N.** A decrease in soil and plant *δ*^15^N generally accompanies increasing MAP and decreasing mean annual temperature (MAT) [11, 80] for MAT ≥ −0.5°C. This pattern may be related to changes in the rate and nature of soil and plant N cycling and/or dependence on mycorrhizal association [11]. Changes in amount of rainfall and soil water availability can affect the openness of the N cycle [81]. A more open N cycle in drier sites probably reflects greater N availability because of lower plant N demand; this can stimulate NH_4_^+^ volatilization, leading to higher soil and plant *δ*^15^N [49, 81]. Lower N availability [82] and greater plant reliance on mycorrhizal association for N acquisition [11] can contribute to lower soil and plant *δ*^15^N in wetter ecosystems. In short, N cycling in ecosystems is highly responsive to climatic factors, and the associated changes in plant *δ*^15^N can be traced from primary producers to consumers [6, 83].

## 2 Materials and methods

### 2.1 Sample collection and preparation

A total of 79 terrestrial plant samples and 15 soil samples (8 topsoil and 7 subsoil including two soil profiles) were collected during September and August 2012 and 2013 (Fig 1) with permission of the Government of Yukon and agreement of Yukon First Nations (Licenses No. 13-52S&E and No. 14-46S&E). Sample collection and field work did not involve any endangered or protected species. Plant and soil samples were placed in woven poly bags and plastic bags, respectively. At sites S13-8 and S13-10, several topsoil samples were collected in response to observed differences in soil texture and topography (shallow *vs*. steep slopes).

All plant samples were air-dried and separated into different plant parts. These plant tissues were washed with distilled water (DW) and dried at 90°C overnight. The dried plant materials were then ground to a very fine powder using a Crescent Wig-L-Bug and stored in small, sealed glass vials for N and C elemental and isotopic measurements.

All soil samples were air-dried, sieved (<2 mm), ground gently using a metal mortar and pestle, and then stored in plastic containers. Analysis of soil samples for physical and chemical properties (mineral fraction, OM fraction, pH, mineralogy) followed standard methods (see Supporting Information: Section A in S1 Text).

Two methods, (i) acid fumigation [84], and (ii) acid rinsing [85], were used to remove carbonates from soil samples prior to elemental and isotopic analyses of OC. Untreated soil samples were used to determine total nitrogen (TN), total carbon (TC), and *δ*^15^N [84].

### 2.2 Elemental analysis

Procedures used for elemental analysis are described in Section B of S1 Text.

### 2.3 Stable isotope analyses

All C and N isotopic results are presented using *δ*-notation [86]:

$$\delta^{13}\text{C or }\delta^{15}\text{N }\left(‰\right)=\left[\left({\text{R}}_{\text{Sa}}/{\text{R}}_{\text{Std}}\right)–1\right]$$ (1)

where R_Sa_ and R_Std_ denote ^13^C/^12^C or ^15^N/^14^N of the sample and standard for *δ*^13^C and *δ*^15^N, respectively. The *δ*-values of all samples were calibrated to VPDB (carbon) and AIR (nitrogen) using USGS40 and USGS41 [87, 88].

The δ^13^C and *δ*^15^N of plant samples, soil OC (after carbonate removal), and soil TN were measured by dry combustion using an EA (Costech Analytical Technologies, Valencia, CA, USA) coupled in continuous flow mode to either a Thermo Scientific Delta^PLUS^ XL or a Thermo Scientific Delta V^PLUS^ IRMS (Thermo Scientific Bremen, Germany). Because of the low N content of the plant samples, nitrogen isotopic analysis was performed in a separate analytical session from that of carbon; CO_2_ generated in the latter sessions was scrubbed from samples using a Carbo-Sorb trap on the EA. Separate analytical sessions were also used to obtain *δ*^15^N for soil TN, using un-acidified samples.

Accuracy and precision of the isotopic analyses were monitored using the laboratory keratin and IAEA-CH-6 (sucrose) standards. The average *δ*^13^C obtained for keratin was –24.05 ± 0.09 ‰ (n = 96), which compares well with its accepted value of –24.04 ‰. The average *δ*^13^C obtained for IAEA-CH-6 was –10.46 ± 0.10 ‰ (n = 33), which compares well with its accepted value of −10.45 ± 0.03 ‰ [87]. Reproducibility for sample duplicates was ± 0.10 ‰ for *δ*^13^C (n = 39). The average *δ*^15^N of keratin was +6.38 ± 0.21 ‰ (n = 108), which compares well with its accepted value of +6.36 ‰. Reproducibility for sample duplicates was ± 0.12 ‰ for *δ*^15^N (n = 27).

### 2.4 Statistical analysis

All plant samples were categorized into two main functional groups: herbs (including annual and perennial grasses, forbs and sedges), and shrub/subshrubs. An independent-sample t-test was used to test for differences in the C and N isotopic and elemental compositions between: (i) different plant functional groups, and (ii) samples from two sampling years (2012 and 2013). Comparisons of C and N isotopic and elemental compositions for (i) different plant parts, and (ii) plants from different sampling sites were performed using one-way ANOVA followed by means comparison using either Tukey’s HSD test, if variance was homogeneous, or Dunnett’sT3 test, if variance was not homogeneous. Assessments of correlation between (i) plant *δ*^13^C and *δ*^15^N, (ii) plant N content and *δ*^15^N, and (iii) soil *δ*^13^C_OC_ and OC in soil profiles were performed using Pearson correlation coefficient. All statistical analyses were performed in SPSS 20.

## 3 Results

### 3.1 Soils

General information for each site sampled is presented in Table 2.

**Table 2:** Environmental data for sampling sites and number of soils and plants sampled.

Site ID	Site Name	Latitude	Longitude	Altitude (masl)	Topography	Plant Samples	Soil Samples
**2012**							
**S12-1**	Chinook Lane, Whitehorse	60.5938	−134.8949	728	-	2	-
**S12-2**	Riverdale, Whitehorse	60.7056	−135.0343	646	Top of a steep slope	7	-
**S12-3**	Carcross Road	60.6213	−135.0169	782	Steep slope	5	-
**S12-4**	Schwatka Lake, Yukon River	60.6724	−135.0250	678	-	3	-
**S12-5**	Miles River Canyon, Yukon River	60.6614	−135.0281	684	Flat, adjust to a highway	7	-
**S12-6**	Destruction Bay	61.4836	−138.3666	844	Flat	7	-
**2013**							
**S13-2**	Cultus Bay, Kluane Lake	61.3850	−138.5220	783	South facing slope (~35–40°)	8	-
**S13-3**	Kluane Lake	61.2581	−138.6161	839	South facing slope (~30–40°)	11	-
**S13-4**	Kluane Lake	61.2461	−138.5650	831	Flat	4	-
**S13-5**	Research Centre, Kluane Lake	61.1836	−138.4008	781	Flat	1	-
**S13-6**	Kluane Lake	61.3530	−138.6178	838	Road cut with SW facing slope (~25°)	8	SP^a^ (1)
**S13-7**	Kluane Lake	61.2530	−138.6019	829	Road cut with NE facing slope (~20°)	6	TS^b^ (1)
**S13-8**	Kluane Lake	61.3092	−138.4458	821	Flat	2	SP (1), TS (2)
**S13-9**	Kluane Lake	61.3413	−138.6419	813	Slope (~10°)	-	TS (1)
**S13-10**	Kluane Lake	61.3039	−138.6611	805	Top & bottom of a steep slope	1	TS (2)
**S13-11**	Mount Decoli, Kluane Lake	61.0514	−137.9867	1138	Steep slop	4	-
**S13-13**	Robert Campbell Highway, Faro	62.1811	−133.7603	748	-	1	-
**S13-14**	North Canol Road, Faro	61.9972	−132.3792	683	-	1	-
**S13-15**	Faro	62.3799	−133.3907	1651	-	1	-
**-**	Slims River	61.0016	−138.5104	-	Flat	-	TS (1)

^a^ SP: soil profile

^b^ TS: topsoil

Seven topsoils, two soil profiles and one topsoil sample of the loess source area (Slims River delta) were analyzed for basic physical and chemical properties (see Supporting Information: Section C and Tables A and B in S1 Text), and OC and TN isotopic compositions (Table 3).

**Table 3:** Isotopic compositions of soil TN and OC.

Sample ID	*δ*^15^N_TN_ (‰, AIR)	*δ*^13^C_OC_ (‰, VPDB)	*δ*^13^C_OC_ (‰, VPDB)
		Acid-fumigated	Acid-rinsed
**S13-6 (0–20)**	+4.3	−24.5	−24.7
**S13-6 (20–30)**	+6.9	−24.3	−24.1
**S13-6 (30–40)**	+7.2	−23.8	−23.7
**S13-6 (40–45)**	+7.0	−23.7	−23.8
**S13-6 (45–60)**	+6.7	−23.8	−23.6
**S13-6 (60–70)**	+4.1	−22.5	−23.0
**S13-7 (0–10)**	+4.0	−24.9	−24.8
**S13-8-1 (0–10)**	+4.6	−25.0	−24.4
**S13-8-1 (10–20)**	+4.2	−24.9	−25.0
**S13-8-1 (20–60)**	+5.3	−24.4	−24.5
**S13-8-2 (0–10)**	+4.8	−24.5	−24.4
**S13-9 (0–10)**	+2.1	−25.1	−23.4
**S13-10-1 (0–10)**	+5.5	−24.5	−24.5
**S13-10-2 (0–10)**	+2.5	−25.2	−25.1
**Slims River**	+1.6	−20.6	−7.5

All topsoils, except for S13-9, are dominated by silt (avg. 48.9 ± 16.5 wt. %, all ± errors reported hereafter are one standard deviation (SD)). The mineralogy of most soil samples is similar to that of Slims River deltaic sediment, which is representative of the sources of windblown deposits in the area (see Supporting Information: Table B in S1 Text).

The total nitrogen isotopic compositions (*δ*^15^N_TN_) of the topsoils range from +2.1 to +5.5 ‰. The *δ*^13^C_OC_ results derived from both types of pretreatment to remove carbonate are very similar except for samples S13-9 and Slims River for which acid fumigation showed more efficacy (Table 3). Accordingly, only results produced using acid fumigation are considered further. The range of *δ*^13^C_OC_ (–25.2 to –24.5 ‰) obtained for topsoils is much smaller than measured for *δ*^15^N_TN_, and is characteristic of C_3_ vegetation. Slims River sediment, by comparison, has *δ*^15^N_TN_ and *δ*^13^C_OC_ of +1.6 ‰ and –20.6 ‰, respectively.

The *δ*^13^C_OC_ of profile S13-6 increases with depth from –24.5 ‰ for topsoil to –22.5 ‰ in the subsoil (Fig 2a). The change in pH with depth in profile S13-6 strongly correlates with inorganic carbon (IC) content, which was calculated by subtracting OC from TC (r = 0.960, p <0.01). The *δ*^15^N_TN_ in profile S13-6 also show a positive shift from +4.3 to +6.7 ‰ with increasing depth from topsoil to 60 cm, but then decreases to +4.1 ‰ between 60–70 cm (Fig 2a). A positive, albeit smaller, shift in both *δ*^13^C_OC_ (–25.0 to –24.4 ‰) and *δ*^15^N_TN_ (+4.6 to +5.3 ‰) with increasing depth is also observed for the second soil profile (S13-8) (Fig 2b).

### 3.2 Plants

#### 3.2.1 Isotopic compositions

A total of 31 (September 2012) and 48 (August 2013) C_3_ plants representing 15 species (*Artemisia frigida*, *Betula glandulosa*, *Bromus pumpellianus*, *Carex filifolia*, *Calamagrostis purpurascens*, *Elymus spicatus*, *Elymus trachycaulus*, *Festuca altaica*, *Linum lewisii*, *Lepidium ramosissimum*, *Plantago canescens*, *Poa glauca*, *Penstemon gormanii*, *Rubus idaeus*, *Salix arctica*) were sampled from 18 sites distributed for the most part just east of Kluane Lake, but also including some locations near the City of Whitehorse and in the Faro area, Yukon Territory (Fig 1, Table 2). The samples represent two main functional groups: herbs (including both annual and perennial grasses, forbs and sedges) (n = 66) and shrub/subshrubs (n = 13) [89]. Subshrubs are smaller than shrubs, but still have a woody base and bushy shape; their soft stems die back during cold seasons.

The *δ*^13^C and *δ*^15^N of leaves (L) and stems (S) were measured for all samples, with additional plant parts analyzed as available (70 root crowns (RC), 68 fine roots (FR), 73 inflorescences (I)) (see Supporting Information: S1 Table). In this study, the root crown is considered as the top part of the root system in herbs and subshrubs. Subshrubs and perennial grasses regrow each spring from buds produced by the root crown. In the herbs, the major vascular changes required for formation of new stems in spring occur in the root crown [90].

Foliar *δ*^13^C for all sampled plants ranges from –30.9 to –25.5 ‰, with a mean of –28.0 ± 1.3 ‰ (n = 79). Foliar *δ*^15^N ranges from –8.3 to +7.0 ‰, with a mean of –0.6 ± 2.7 ‰ (n = 77). The foliar *δ*^15^N of two samples (*A*. *frigida*, Site: S13-2, year: 2013), which are extremely high (+23.4 and +18.7 ‰), have been excluded from this range and subsequently reported averages. Intense volatilization (ε = 40–60 ‰) at the soil surface resulting from urine/dung fertilization by herbivores at this spot could have caused such extreme ^15^N-enrichment [91].

No statistically significant differences in foliar *δ*^13^C and *δ*^15^N are observed between the two functional groups (*δ*^13^C: t(14.08) = 1.30, p >0.05; *δ*^15^N: t(75) = 0.81, p >0.05), although shrubs and subshrubs have slightly higher mean foliar *δ*^15^N than herbs (+0.1 ‰, n = 11) *vs*. (−0.6 ‰, n = 66). Within a given plant, there is a clear pattern of lower foliar *δ*^13^C relative to all other plant parts, as expressed by the difference in plant part *vs*. leaf *δ*^13^C (*Δ*^13^C_plant part-leaf_) (see Supporting Information: S1 Fig); Average values of all individual samples are as follow: *Δ*^13^C_RC-L_ = 0.5 ± 0.8 ‰, *Δ*^13^C_FR-L_ = 0.6 ± 0.8 ‰, *Δ*^13^C_S-L_ = 0.9 ± 1.1 ‰, *Δ*^13^C_I-L_ = 0.6 ± 1.4 ‰. In contrast, no clear pattern of intra-plant variation is observed for *δ*^15^N (see Supporting Information: S2 Fig); Average values of all individual samples are as follow: *Δ*^15^N_RC-L_ = 0.2 ± 2.5 ‰, *Δ*^15^N_FR-L_ = −0.1 ± 2.9 ‰, *Δ*^15^N_S-L_ = 0.1 ± 1.6 ‰, *Δ*^15^N_I-L_ = 0.3 ± 1.8 ‰. The range of intra-plant variation in *δ*^13^C is much smaller than measured for *δ*^15^N (~4 *vs*. 12 ‰).

**Fig 3:**
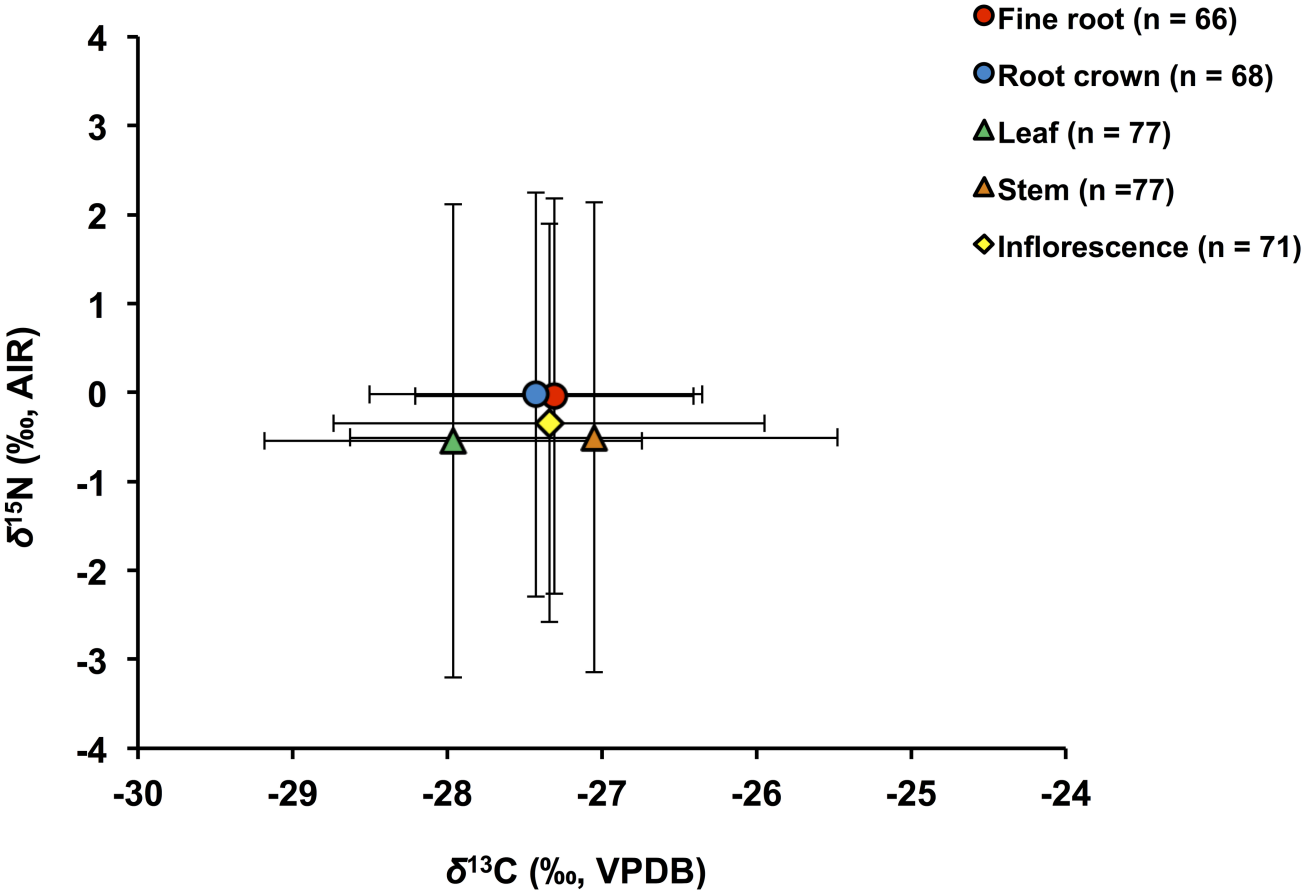
Mean (± SD) *δ*^13^C and *δ*^15^N of all plant parts analyzed, including fine root, root crown, leaf, stem and inflorescence. Below-ground plant parts have higher mean *δ*^**15**^N than above-ground parts, and foliar *δ*^**13**^C is lower than that of fine roots, stems and inflorescences, but only the latter is statistically significant (see text for details).

**Fig 4:**
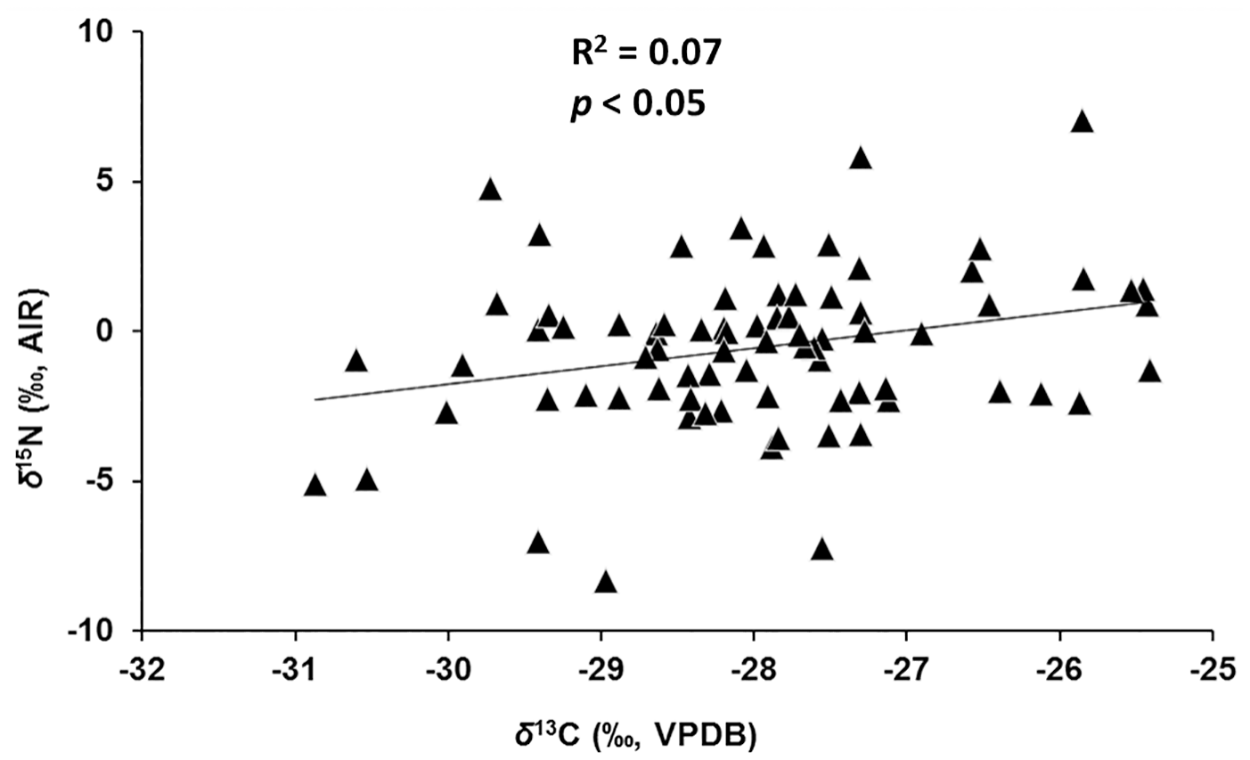
Foliar carbon and nitrogen isotopic compositions of all plant samples. There is a weak, positive correlation between *δ*^13^C and *δ*^15^N (see text).

Mean *δ*^13^C and *δ*^15^N for different plant tissues for all plants are illustrated in Fig 3. A one-way ANOVA comparing different plant parts of all plants (F(4,364) = 5.57, p <0.01) confirms a significant intra-plant variations in *δ*^13^C with foliar *δ*^13^C significantly lower than that of fine roots, stems and inflorescences (Table 4). Below-ground plant parts (RCs and FRs) have higher mean *δ*^15^N than above-ground parts (L, S and I), but the difference is not significant (F(4,355) = 0.73, p >0.05). There is a weak, but statistically significant, positive correlation between foliar *δ*^15^N and *δ*^13^C for all plant samples from all sites (Fig 4). An independent-samples t-test comparing mean 2012 and 2013 foliar *δ*^13^C and *δ*^15^N shows no significant difference between the two years (*δ*^13^C: t(77) = 0.52, p> 0.05 and *δ*^15^N: t(75) = 0.49, p >0.05). A one-way ANOVA comparison of foliar *δ*^13^C and *δ*^15^N among sites with ≥5 samples shows a significant difference in *δ*^13^C_2012_ and *δ*^15^N_2013_ between different sites (*δ*^13^C_2012_: F(3, 22) = 5.55, p <0.01 and *δ*^15^N_2013_: F(3, 27) = 7.79, p <0.01). The results of ANOVA post-hoc test (Tukey’s HSD/Dunnett’s T3) for these comparisons are presented in Table 5. There are statistically significant differences in foliar *δ*^13^C between sites S12-5 and S12-2, and sites S12-5 and S12-6 in 2012, with site S12-5 having a lower foliar mean value (–28.7 ‰) than sites S12-2 (–26.9 ‰) and S12-6 (–27.3 ‰). In 2013, there are statistically significant differences in foliar *δ*^15^N between sites S13-6 and S13-7, sites S13-6 and S13-3 and sites S13-6 and S13-2, with site S13-6 having a higher foliar mean values (+1.4 ‰) than sites S13-7 (−2.0 ‰), S13-3 (–1.3 ‰) and S13-2 (−0.74 ‰).

**Table 4:** ANOVA post-hoc tests (Dunnett’s T3) results for *δ*^13^C differences among plant tissues.

	*δ*^13^C (‰, VPDB)			
**Plant Part**	**FR**^a^	**I**^b^	**L**^c^	**RC**^d^
**S**^e^	0.951	0.970	**0.001**	0.654
**RC**	0.997	1.000	**0.048**	-
**L**	**0.002**	**0.024**	-	-
**I**	1.000	-	-	-
**FR**	-	-	-	-

^a^ Fine root,

^b^ Inflorescence,

^c^ Leaf,

^d^ Root crown,

^e^ Stem.

Values in boldface font are statistically significant (p ≤0.05).

**Table 5:** ANOVA post-hoc tests (Tukey’s HSD) for foliar isotopic differences between sites (plants sampled ≥5).

	***δ*^13^C (‰, VPDB)**		
**Sites (2012)**	**S12-6**	**S12-5**	**S12-3**
**S12-2**	0.855	**0.004**	0.490
**S12-3**	0.892	0.188	-
**S12-5**	**0.027**	**-**	-
	***δ*^15^N (‰, AIR)**		
**Sites (2013)**	**S13-7**	**S13-6**	**S13-3**
**S13-2**	0.471	**0.050**	0.854
**S13-3**	0.821	**0.002**	-
**S13-6**	**0.001**	-	-

Values in boldface are statistically significant (p ≤0.05).

#### 3.2.2 Carbon and nitrogen contents

**Fig 5:**
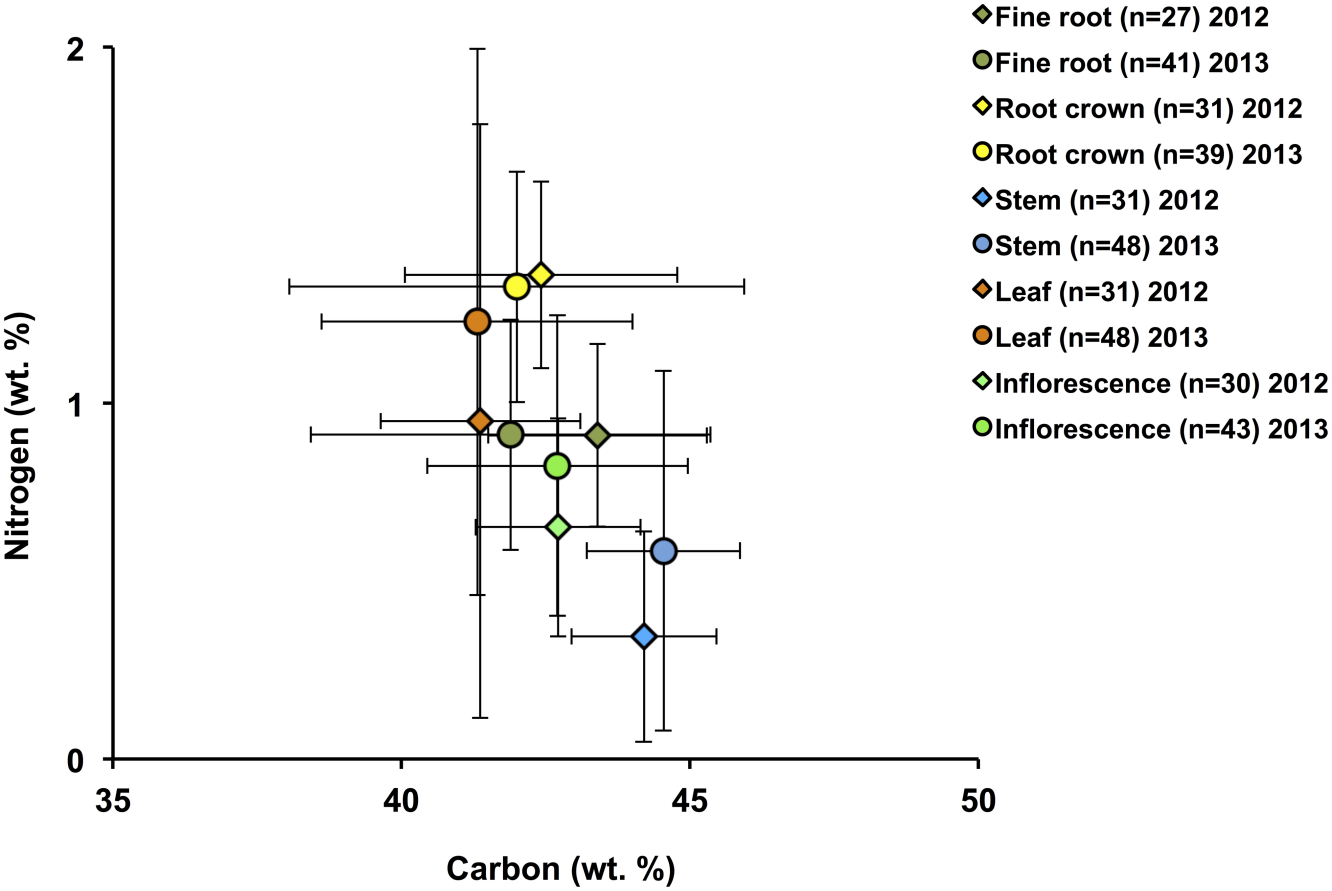
Average N and C contents of plant parts according to sampling year. In both 2012 and 2013, root crowns have the highest N content whereas stems have the lowest (see text); likewise, during both years, carbon contents are highest in stems, and lowest in leaves.

Foliar N contents range from 0.3 to 4.0 wt. %, with an average of 1.1 ± 0.8 wt. % (n = 79). Foliar C contents range from 34.7 to 48.0 wt. %, with an average of 41.3 ± 2.4 wt. % (n = 79) (Supporting Information: S2 Table). Foliar atomic C/N ranges from 13.3 to 156.7, and averages 59.8 ± 30.9 (Supporting Information: S2 Table). In both 2012 and 2013, root crowns have the highest N content (avg. 1.3 ± 0.3 wt. %, n = 70), and stems the lowest (avg. 0.5 ± 0.5 wt. %, n = 79) (Fig 5). A one-way ANOVA comparison of C and N contents between different plant parts shows a significant difference (C: F(4, 364) = 16.28, p <0.01 and N: F(4, 364) = 33.77, p <0.01); Table 6 summarizes the results of ANOVA post-hoc tests (Dunnett’s T3) between different plants parts.

**Table 6:** ANOVA post-hoc tests (Dunnett’s T3) for differences in C and N contents between different plant parts.

	C (wt. %)				N (wt. %)			
**Plant Part**	**FR**^a^	**I**^b^	**L**^c^	**RC**^d^	**FR**	**I**	**L**	**RC**
**S**^e^	**0.000**	**0.000**	**0.000**	**0.000**	**0.000**	**0.002**	**0.000**	**0.000**
**RC**	1.000	0.943	0.551	-	**0.000**	**0.000**	0.209	-
**L**	0.111	**0.001**	-	-	0.263	**0.004**	-	-
**I**	1.000	-	-	-	**0.043**	-	-	-

^a^ Fine root,

^b^ Inflorescence,

^c^ Leaf,

^d^ Root crown,

^e^ Stem.

Values in boldface font are statistically significant (p ≤0.05).

On average, foliar C and N contents of shrubs and subshrubs (avg. C (wt. %) = 44.5, avg. N (wt. %) = 2.1) are significantly higher than herbs (avg. C (wt. %) = 40.7, avg. N (wt. %) = 0.9) (Independent-samples t-test: C: t(77) = 6.46, p <0.01; N:t(13.47) = 4.13, p <0.01). A statistically significant positive correlation is observed between foliar *δ*^15^N and N (wt. %) contents among all plants analyzed (r = 0.389, p <0.01).

## 4 Discussion

### 4.1 Kluane Lake Soils

All topsoil samples examined along the eastern shoreline of Kluane Lake are rich in silty eolian sediment that likely originated from the Slims River delta [21, 24] except for S13-9, which contains a larger abundance of sand (Supporting Information: Table A in S1 Text). Its higher sand content likely reflects a larger contribution of underlying sandy glaciofluvial deposits resulting from bioturbation or post-fire redistribution [24]. The soil mineralogy is similar to the Slims River sediment, consistent with an eolian source (Supporting Information: Table B in S1 Text).

**Fig 6:**
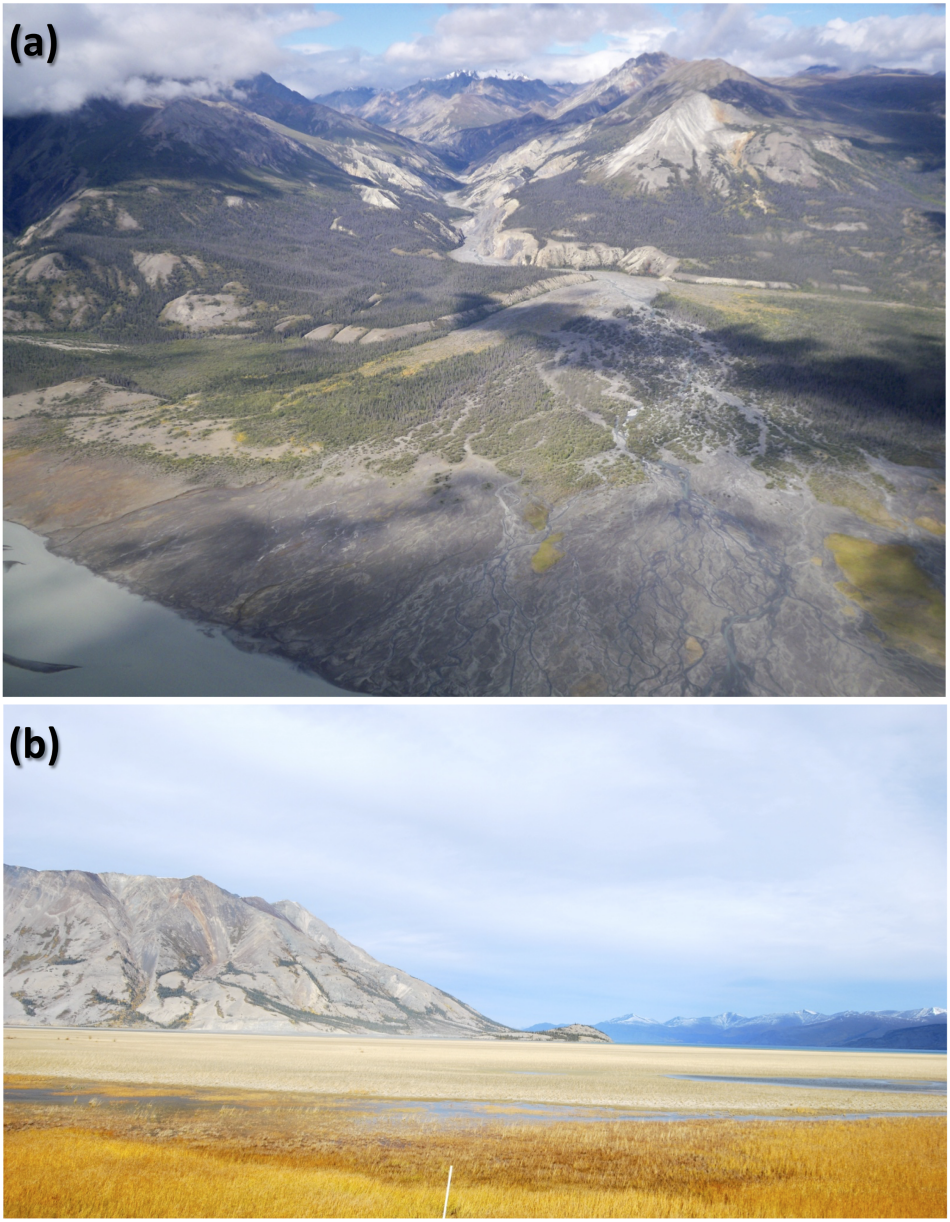
(a) Areal view of the Slims River delta; (b) Slims River vegetation in August. (photographic credits: Fred Longstaffe and Tessa Plint).

The average *δ*^13^C_OC_ (–24.8 ± 0.3 ‰) of all topsoils is typical of soil organic matter generated by C_3_ vegetation, except for the higher *δ*^13^C_OC_ (–20.6 ‰) of the Slims River deltaic sediment. This site (Fig 6), which is one of the two main outlets of the Kaskawulsh glacier [92], is subject to flooding and seasonal and diurnal fluctuations in the water flow, and supports algae and macrophytes growth at low flow stages. Aquatic plants in Yukon Territory have a wide range of foliar *δ*^13^C_OC_ (−41 to −15 ‰), particularly for submerged macrophytes [93]. Less negative *δ*^13^C_OC_ (−16 to −13 ‰) for aquatic plants and algae have also been reported for Arctic continental shelf sediments [94]. Contributions of CAM or C_4_ plants at modern, northern high-latitude site like this one are unlikely [39]. Incomplete removal of carbonate can also be ruled out based on the acid fumigation results (Table 3).

**Fig 7:**
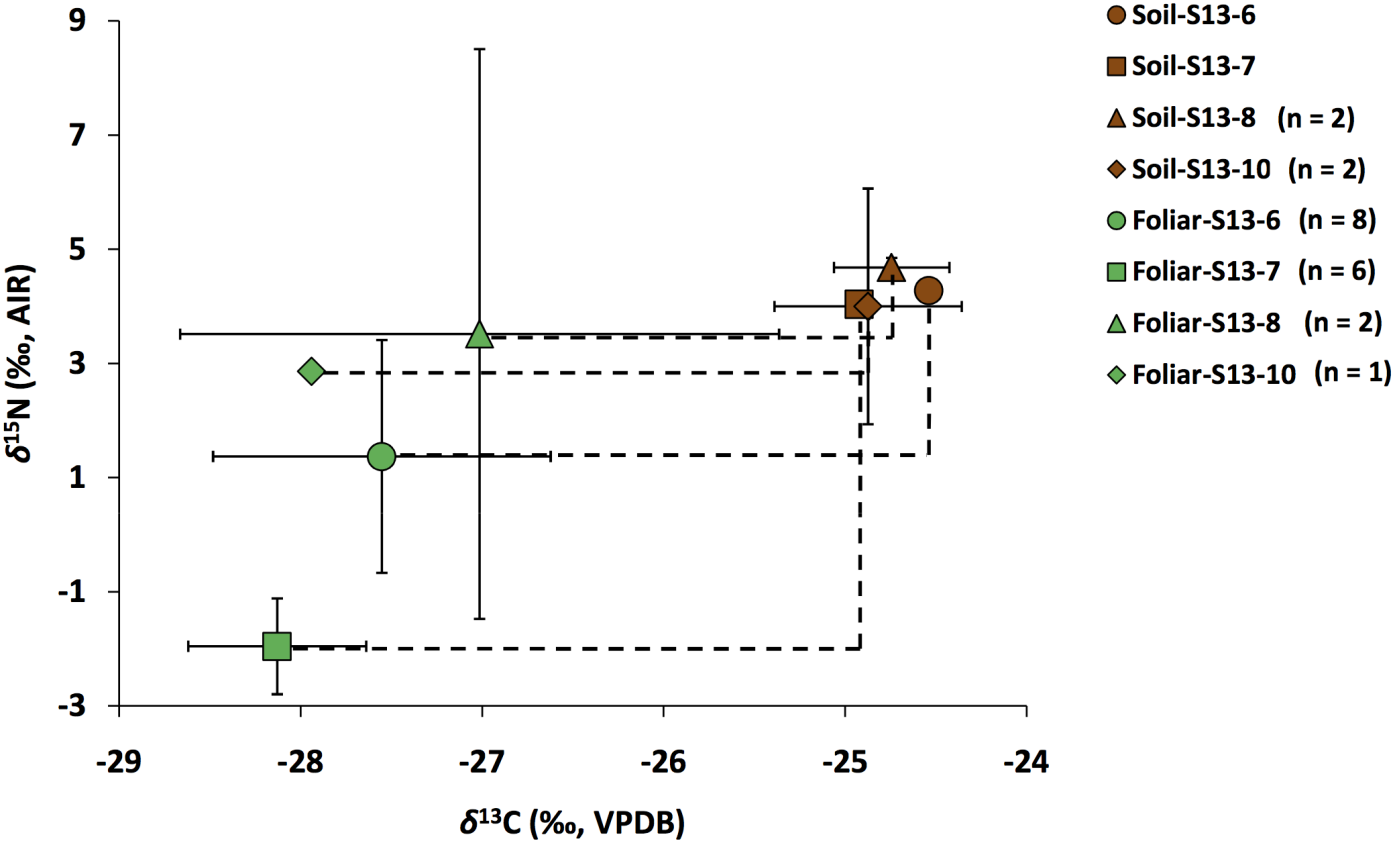
Comparison of topsoil and foliar average (± SD) C and N isotopic compositions. Smaller site-to-site variations in topsoil *δ*^15^N_TN_ relative to foliar *δ*^15^N likely reflect a combination of soil processes and plant sample size and species (see text).

In general, the topsoil samples have higher average *δ*^15^N_TN_ and *δ*^13^C_OC_ than plants from the same sites (Fig 7), which is typical of many other terrestrial ecosystems [16, 71]. This can be explained by the general enrichment in ^13^C and ^15^N of plant tissues in soil during organic degradation [12, 32]. The larger isotopic variation among the foliar isotopic compositions relative to topsoil samples likely reflects differences in plant sample size, number of plant species sampled, and the age of plants at different sites. In particular, enzymatic variability among different plant species and different patterns of N acquisition among co-occurring species [72] serves to increase the range of foliar *δ*^15^N among sites and between plants within sites and their topsoil. In contrast, the continuous activities of soil decomposers (mainly fungi and bacteria), the strong role of soil minerals in stabilizing soil chemistry [95] and the open N dynamics of soil input and output reactions reduce N isotopic variations in topsoil from site to site within the same general region.

The increase in *δ*^13^C_OC_ with depth in two soil profiles (Fig 2a and 2b) is in agreement with earlier results for subarctic ecosystems [27, 96]. This pattern likely reflects the input of fresh litter with lower *δ*^13^C at the soil surface and accumulation of decomposed and hence more ^13^C-rich OM at greater depths [27, 33]. This interpretation is consistent with the strong negative correlation between *δ*^13^C_OC_ and OC content (r = −0.950, p <0.05) and the decrease in OC content with depth (Fig 2a and 2b).

The change in *δ*^15^N_TN_ with depth observed in both soil profiles (Fig 2a and 2b) has been reported previously for such soils from Siberia [27]. The negative shift in *δ*^15^N_TN_ below 60 cm in profile S13-6 may arise from the very small amount of OM (2.9 wt. %) and TN (0.1 wt. %) at this depth, in which case the contribution of inorganic nitrogen to the *δ*^15^N_TN_ signal may be more important at this depth. In soils, organic N typically has higher *δ*^15^N (+5 to +7 ‰) than inorganic forms (NO_3_^-^ and NH_4_^+^) (−2 to +5 ‰) [12, 56].

Values of *δ*^15^N_TN_ are positive in all topsoil samples, which is typical of alpine and tundra ecosystems [27, 53]. Soil *δ*^15^N_TN_ >0 ‰ point to inputs with compositions higher than those produced by fixation of nitrogen from air and/or N-loss processes that leave the soil N pool enriched in ^15^N [97]. Two samples (S13-9, S13-10-2) sit at the lowest end of the N isotopic range (+2.1 to +5.5 ‰). S13-9 has the highest sand and lowest clay and silt contents of the soils examined in this study. The content, structure and function of OM associated with mineral particles generally vary with grain size. Sand-sized particles are typically associated with less humified and lower abundances of OM than silt and clay [95]. Several studies of soils underlying grasslands or forest have reported higher *δ*^15^N_TN_ for clay-sized fractions (~+9 to +12 ‰), which generally have a higher content of stable, humified OM than silt (+5 to +9 ‰) and sand (+2 to +7 ‰) particles [98–101].

Topography may explain the low *δ*^15^N_TN_ (+2.5 ‰) of sample S13-10-2 at site S13-10. Sample S13-10-2 was collected from top of a steep slope, which made it more susceptible to erosional disturbance, while sample S13-10-1 (*δ*^15^N_TN_ = +5.5 ‰) (from the same site) was collected at the bottom of the slope from a flat and more stable location. Steeply sloping soils can have *δ*^15^N close to atmospheric inputs because of continuous soil removal and soil organic matter rejuvenation, which maintains the soil’s N status far from steady state [80].

### 4.2 Plant C and N isotopic and elemental compositions

**Fig 8:**
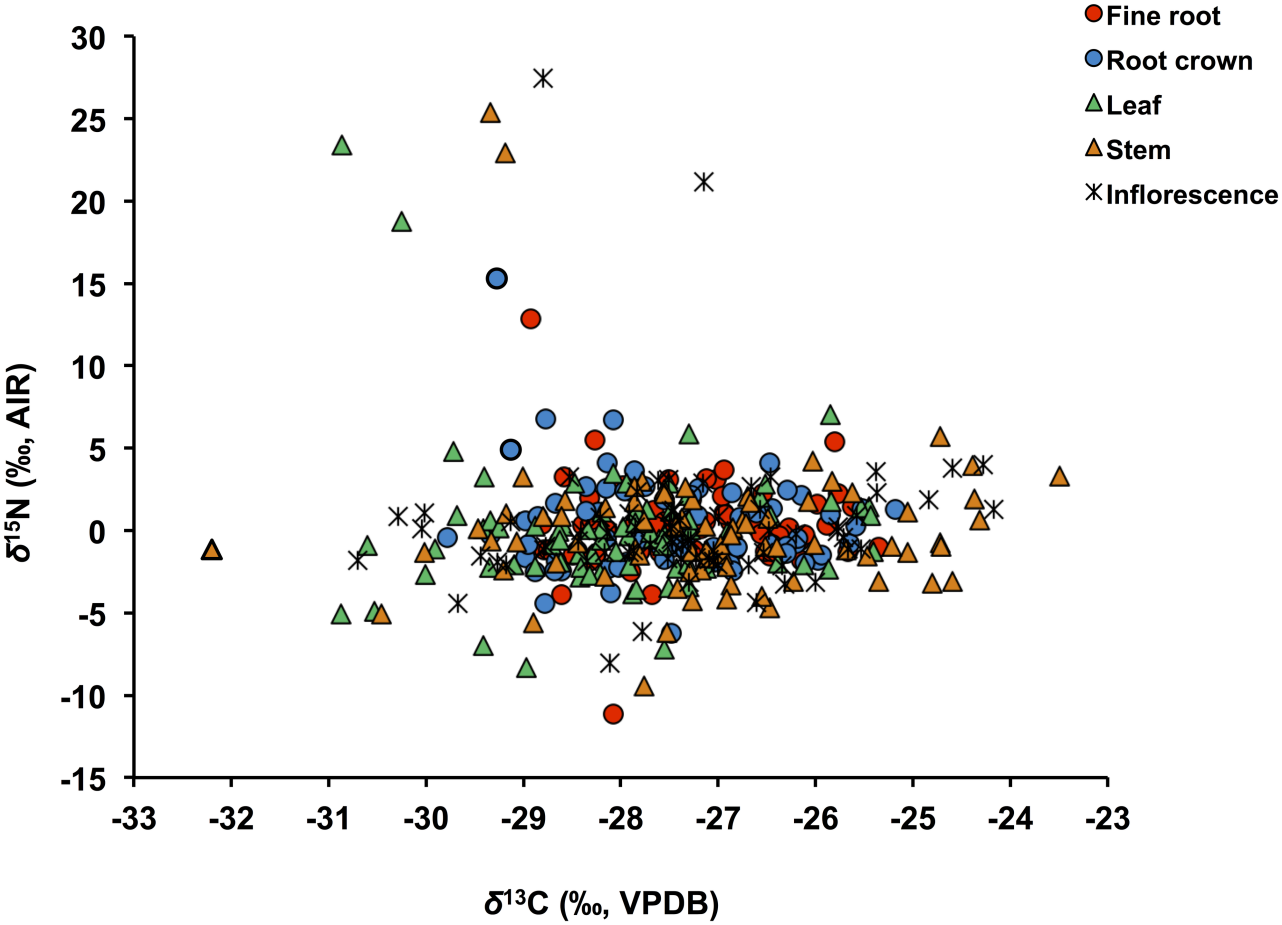
Nitrogen versus carbon isotopic compositions of fine root, root crown, leaf, stem and inflorescence. The wide spread in *δ*^15^N is typical of nitrogen-limited ecosystems, and utilization of a range of soil nitrogen sources (see text).

The range of *δ*^13^C (−32.5 to −23.5 ‰) measured for all plant parts in this study (Fig 8) is typical of C_3_ vegetation, which dominates high latitude ecosystems [39]. Variation in environmental factors (slope aspect, light, water availability and topography) even within small microhabitats may cause this large spread in *δ*^13^C. Wooller et al. [14] reported a very similar range of foliar *δ*^13^C for sedges and grasses from Alaska and Yukon Territory.

The plants analyzed here show great variation in *δ*^15^N, spanning ~ 40 ‰ (Fig 8). Variation in *δ*^15^N of vegetation tends to be more pronounced in N-limited ecosystems, which is typical of Arctic and subarctic regions, and points to utilization of different soil N resources by plants depending on their life forms, type of mycorrhizal association and rooting depth and morphology [16]. Coexisting plant species are known to partition N resources with different *δ*^15^N in these ecosystems [16, 17, 56, 102]. For example, the grass *Calamagrostis canadensis* (*δ*^15^N = +0.9 ‰) in Alaska acquires N from deeper soil horizons, while the evergreens *Picea glauca* and *Picea mariana* (−7.7 ‰) likely utilize ammonium or organic N from fresh litter [17].

There is no statistically significant difference in foliar *δ*^13^C and *δ*^15^N between herbs and shrubs/subshrubs in this study. Considering that all plants studied here utilize the C_3_ photosynthetic pathway, the lack of any clear distinction in foliar *δ*^13^C is not surprising. The absence of systematic differences in *δ*^15^N between these two groupings is less expected, given potential differences in N conservation and resorption efficiency [103] and patterns of root distribution and rooting depth, which affect resource acquisition [104]. The absence of a statistically meaningful difference in *δ*^15^N might arise from the unequal sample size of herbs *vs*. shrubs/subshrubs or confounding environmental factors such as plant growth stage, the range of available N sources, and the varied environmental conditions in the study area.

The shrubs and subshrubs have statistically significantly higher C and N contents than the herbs at both foliar and whole plant levels, as has been observed previously, especially for N [104]. The difference in N content measured here might be related to different N conservation strategies during the late growing season. Arid and semi-arid perennial grasses have lower N contents in senesced leaves and overall higher N conservation efficiency than shrubs, which is an important adaptive trait for plants from nutrient-limited ecosystems [103]. Plants in this study were sampled in very late growing season (mid-late September in 2012 and late August in 2013), with perennial grasses comprising most herb samples. Hence, the difference in N contents may indicate higher N resorption efficiency from senescing tissues in grasses than in shrubs.

There are likely four main environmental factors that control the isotopic compositions of the herbaceous plants, shrubs and subshrubs sampled in this study: (i) water availability, (ii) N availability, (iii) spacial differences, and (iv) intra-plant variation. Each factor is discussed next.

#### 4.2.1 Water availability

The very weak, but statistically significant positive correlation between foliar *δ*^15^N and *δ*^13^C of all samples analyzed (Fig 4) may point to water availability as at least a minor factor affecting both *δ*^15^N and *δ*^13^C of plants in this ecosystem. Plants capture atmospheric CO_2_ through leaf stomata and fix it using enzymatic reactions, while N is mainly obtained through roots from soil or symbiotic associations and then assimilated. Given that the sources and pathways determining the *δ*^13^C and *δ*^15^N of plants are different, the observed, albeit weak, correlation may indicate a common environmental factor affecting both isotopic signals. As noted earlier, a change in MAP can affect both *δ*^13^C and *δ*^15^N of plants in the same direction (N: [11, 54, 80]; C: [42, 105, 106]). Ma et al. [43], for example, have reported an aridity-associated positive correlation between *δ*^13^C and *δ*^15^N in plants from northern China and noted that plant isotopic sensitivity to water availability can vary among ecosystems and plant species. Such variations warrant further consideration in paleoecological and paleodietary reconstructions of Arctic and subarctic regions.

#### 4.2.2 N availability

**Fig 9:**
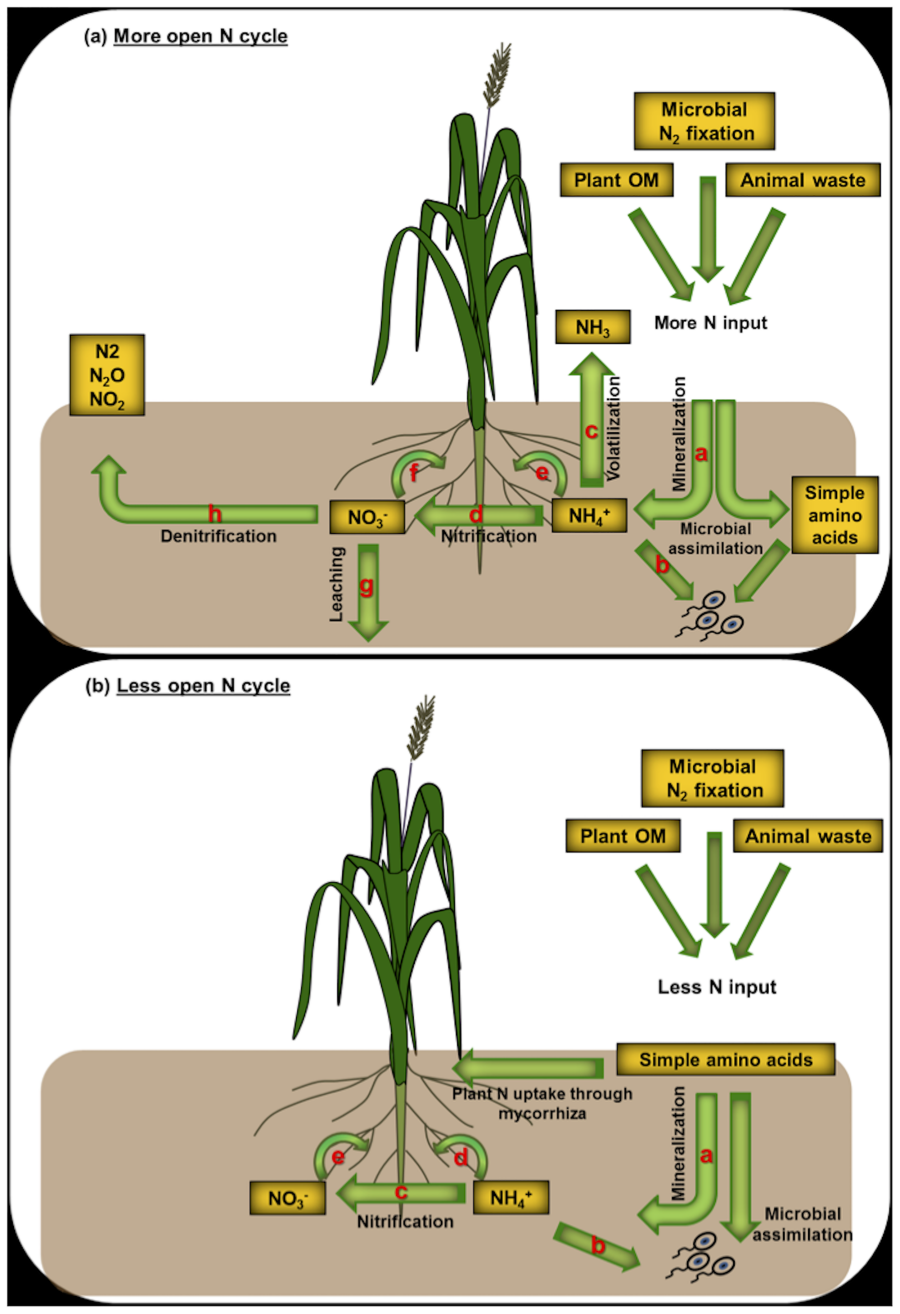
A simplified model for the “openness” of the N cycle in ecosystems with high (9a) and low (9b) N availability. (**9a**): (a) N mineralization: Conversion of organic N to NH_4_^+^ (ε = 0–5 ‰); (b) Microbial assimilation: incorporation of NH_4_^+^ into microbial biomass (ε = 14–20 ‰); (c) NH_3_ volatilization: conversion of NH_4_^+^_(aq)_ to NH_3(g)_ (ε = 40–60 ‰); (d) Nitrification: conversion of NH_4_^+^ to NO_3_^-^ (ε = 15–35 ‰); (e) Plant uptake and assimilation of NH_4_^+^ (ε = 9–18 ‰); (f) Plant uptake and assimilation of NO_3_^-^ (ε = 0–19 ‰); (g) NO_3_^-^ leaching (ε = 0–1 ‰); (h) Denitrification: conversion of NO_3_^-^ to N_2_O, N_2_ and NO_2_ (ε = 28–33 ‰).(**9b**): (a) N mineralization: Conversion of organic N to NH_4_^+^ (ε = 0–5 ‰); (b) Microbial assimilation: incorporation of NH_4_^+^ into microbial biomass (ε = 14–20 ‰); (c) Nitrification: conversion of NH_4_^+^ to NO_3_^-^ (ε = 15–35 ‰); (d) Plant uptake and assimilation of NH_4_^+^ (ε = 9–18 ‰); (e) Plant uptake and assimilation of NO_3_^-^ (ε = 0–19 ‰). Values of ε are from Robinson [50] and Houlton and Bai [51]).

The positive correlation between foliar *δ*^15^N and N content suggests a key role for N availability and N cycling in determining the N isotopic signal acquired by plants in this ecosystem. Such a correlation has been reported previously on local [107], regional [54, 108] and global [11] scales. Higher plant *δ*^15^N reflects higher N availability and a more open N cycle in terrestrial ecosystems [107, 109–111]. The globally observed positive correlation between soil and foliar *δ*^15^N suggest foliar *δ*^15^N as an index for N availability in soils and therefore ecosystems [11]. Nonetheless, the best way to describe N availability for plants in different ecosystems remains unclear [11]. It can be defined in several ways including: (i) an increase in N inputs into the soil from different sources (animal dung, plant materials, microbial N fixation), (ii) increased OM decomposition and N mineralization, (iii) increased NO_3_^−^ production through more nitrification [111], and (iv) less N demand by plants, particularly in drier localities [81]. In any of these scenarios, higher inorganic N availability in soils means that extra N is available to fuel N loss processes (e.g. denitrification and volatilization), which leave the bioavailable N in soils (NO_3_^−^, NH_4_^+^) enriched in ^15^N [49, 50] (Fig 9a). In such ecosystems, plants acquire both higher *δ*^15^N and N content in their leaves, which is characteristic of a more open N cycle. In N-limited ecosystems, by comparison, there is less N-bearing material available for N loss, and hence less opportunity for ^15^N enrichment of the system through such processes. Plants also rely more heavily on mycorrhizal fungi for N acquisition, which is a more ^15^N-depleted source [11, 58] (Fig 9b).

#### 4.2.3 Spacial differences

The statistically significant differences observed in foliar *δ*^15^N among sites S13-2, S13-3, S13-7 and S13-6 points to heterogeneity in this ecosystem and the presence of different microhabitats even at small scales. These differences may be related to local variations in soil properties, slope aspects, topography, water availability, animal disturbance and grazing, which in turn can affect local N cycling. While we might predict higher foliar *δ*^15^N at S13-7 than S13-6 given the former’s higher topsoil OM content (9.2 *vs*. 7.5 wt. %) and TN (0.4 *vs*. 0.2 wt. %), the opposite result is obtained (S13-7-avg. *δ*^15^N = −2.0 ‰, n = 6) *vs*. (S13-6-avg. *δ*^15^N = +1.4 ‰, n = 8). Other factors such as slope aspect and topography may have overshadowed the influence of soil properties. In S13-6, plant samples were collected from north side of the road on a SW-facing shallow slope, while in S13-7, plants were sampled on south side of the road on a NE-facing, shallow slope. This difference influenced the microclimate and vegetation pattern [24], as is indicated by the dominance of subshrubs at S13-7 and herbs at S13-6.

Leaves from S13-6 also have higher *δ*^15^N (avg. +1.4‰, n = 8) than those from site S13-3 (avg.−1.3 ‰, n = 11) and site S13-2 (avg. −0.7 ‰, n = 6). Soil at site S13-6 may provide plants with N pools having higher *δ*^15^N because of its shallower slope than site S13-3, and different topography than site S13-2.

Plants at S12-5 (avg. −28.7 ‰, n = 7) have significantly lower average foliar *δ*^13^C than at S12-2 (avg. −26.9 ‰, n = 7) and S12-6 (avg. −27.3, n = 7). The adjacency of site S12-5 to a main highway may contribute to lower plant *δ*^13^C arising from carbon dioxide additions from vehicle fuel combustion (typically –29.3 to –27.6 ‰; [112]). While Site S12-2 is also located adjacent to a main road, sampling occurred on a steep slope that is substantially elevated from the road.

#### 4.2.4 Intra-plant variations

The average *Δ*^13^C_other plant part-leaf_ measured in this study are positive, consistent with previous studies [46, 113]. The differences measured here for most individual specimens, however, are small (<1 ‰) (Supporting Information, S1 Fig), and should not affect the use of *δ*^13^C to infer diet for animals that may prefer one plant part over another as forage.

No clear pattern in *Δ*^15^N_other plant part-leaf_ was observed, likely because of confounding factors such as differences in root morphology, depth and distribution, microhabitat, mycorrhizal associations and growing stage. The root crowns examined here have higher N contents (Fig 5) and *δ*^15^N (Fig 3) than other plant parts; this difference is statistically significant for N content, but not for *δ*^15^N. As discussed earlier, nitrogen contents of different plant parts commonly show variations between active growing and senescent stages in herbaceous plants [114, 115]. The higher N content of the root crowns in this study is best explained by N re-allocation from leaves to below ground parts late in the growing season.

## 5 Summary and implications

All plants analyzed from this ecosystem follow the C_3_ photosynthetic pathway, and have an average whole plant *δ*^13^C of −27.5 ± 1.2 ‰ and foliar *δ*^13^C of –28.0 ± 1.3 ‰. The plants analyzed here showed very little variability in *δ*^13^C among different plant parts and sampling sites. Their isotopic composition, with suitable correction for the Suess effect arising from fossil fuel combustion since the Late Pleistocene, provides a suitable baseline for interpreting the diet of ancient herbivores that lived in these grasslands.

The nitrogen isotopic data for these modern plants (average whole plant *δ*^15^N = −0.3 ± 2.2 ‰ and foliar = –0.6 ± 2.7 ‰) provide a good baseline for the region’s vegetation under present conditions of nitrogen cycling. The wide range of intra-plant and inter-plant *δ*^15^N variations likely arises from multiple factors, including water availability, N availability and topography. Such variation is likely typical of vegetation from different microhabitats within Arctic and subarctic ecosystems. Given this complexity, modern vegetation *δ*^15^N is unlikely to adequately approximate Late Pleistocene vegetation in the same region.

A growing body of studies utilizes the isotopic composition of bone collagen and other tissues from Late Pleistocene megaherbivores in high latitudes to reconstruct their diet. These works are hampered by insufficient isotopic data for plants at the base of food web in these now vanished ecosystems. More data for both modern and well-dated fossil plants from these ecosystems are needed to fully document and understand the variations in plant isotopic baselines both through time and space. The present study provides a starting point for modern vegetation in south and central Yukon Territory, Canada. While the plant N isotopic compositions presented here cannot be applied directly to the past, it provides a baseline for comparison with its ancient equivalent, which can be obtained by analyzing fossil plants from the same region. We note that the significant positive correlation between foliar δ^15^N and N content observed for this region has potential as an index for forage quality. Comparison of the modern N isotopic vegetation baseline for this region with its Late Pleistocene equivalent could serve as a proxy for tracing changes in forage quality, which is tightly connected to ecosystem productivity and potentially, the sustainability of megaherbivore populations.

## Supporting information

S1 Text Section A. Methods for analyzing physical and chemical properties of soil. Section B. Analysis of carbon and nitrogen content. Section C. Results of soil analysis. Table A: Soil characteristics. Table B: Soil mineralogy.
(DOCX)

S1 Table *δ*^13^C and *δ*^15^N of all plant parts. (DOCX)

S2 Table Carbon and nitrogen contents and foliar atomic C/N of all plants. (DOCX)

S1 Fig Differences in *δ*^13^C between other plant tissues and leaf (*Δ*^13^C). Dashed lines represent means.
(TIFF)

S2 Fig Differences in *δ*^15^N between other plant tissues and leaf (*Δ*^15^N). Dashed lines represent means.
(TIFF)
