# Supplementary material for: Nitrogen and carbon isotopic dynamics of subarctic soils and plants in southern Yukon Territory and its implications for paleoecological and paleodietary studies
Source: PLoS One. 2017 Aug 16;12(8):e0183016. doi: 10.1371/journal.pone.0183016 (PMC5559067; doi:10.1371/journal.pone.0183016)
Supplement: S1 Text — Section A. Methods for analyzing physical and chemical properties of soil. Section B. Analysis of carbon and nitrogen content. Section C. Results of soil analysis. Table A: Soil characteristics. Table B: Soil mineralogy. (DOCX) [file pone.0183016.s001.docx]

1. **Methods for analyzing physical and chemical properties of soil**

Clay, silt and sand fractions [1] were quantified using the hydrometer method [2] after removal of organic matter (OM) using sodium hypochlorite [3]. For particle size analysis, soil particles were dispersed using a probe-type ultrasonic in 0.05 % sodium hexametaphosphate solution. The OM content of soil samples was determined by loss-on-ignition (LOI; 550ᵒC for 4 h [4]). Soil pH was measured in 0.01 M calcium chloride [5]. The mineralogy of soil samples (oven-dried at 65ᵒC) was determined using powder X-ray diffraction (pXRD) using a Rigaku, high brilliance, rotating-anode X-ray diffractometer equipped with a graphite monochrometer and CoKα radiation produced at 45 kV and 160 mA. The abundance of each mineral was estimated using the unweighted, background-subtracted, peak height of its most intense diffraction.

1. **Analysis of carbon and nitrogen content**

Acid-treated and untreated soils were analyzed for OC, TC and TN contents (dry wt. %) by dry combustion using a Fisons 1108 Elemental Analyzer, which was calibrated using High Organic Content Sediment and Low Organic Content Soil. Reproducibility for OC was ± 0.05 wt. % (2 sample duplicates), ± 0.06 wt. % (6 replicates of High Organic Content Sediment) and ± 0.01 wt. % (6 replicates of Low Organic Content Soil). Reproducibility for TN was ± 0.01 wt. % (3 sample duplicates) and ± 0.01 wt. % (6 replicates each of Low Organic Content Soil and High Organic Content Sediment).

The OC and TN contents (dry wt. %) of plant samples were determined using an Elemental Analyzer (EA) (Costech Analytical Technologies, Valencia, CA, USA) coupled to either a Thermo Scientific Delta^PLUS^ XL or a Thermo Scientific Delta V^PLUS^ isotope ratio mass spectrometer (IRMS) (Thermo Scientific, Bremen, Germany). The OC and TN abundances were calibrated using USGS40 and USGS41. Analytical precision and accuracy for OC and TN contents was determined using the internal laboratory standard keratin for C and N and NIST 1547 (Peach Leaves) for N. For some analytical sessions, precision was acceptable (within ± 1 % for C and within ± 0.5 % for N) but accuracy was outside of the acceptable range. For these samples, the results were recalibrated using data from separate analyses performed using a Fisons 1108 EA. Any samples for which both precision and accuracy were outside of acceptable limits (> ± 1 wt. % for C and > ± 0.5 wt. % for N) were reanalyzed using the Fisons 1108 EA. Samples analyzed using the Fisons 1108 EA were calibrated to the laboratory acetanilide standard.

The average C and N contents for the keratin standard were 48.13 ± 0.83 wt. % (n = 66) and 14.46 ± 0.49 wt. % (n = 100), respectively, which compare well with their expected values of 48.22 ± 1.07 wt. % and 14.85 ± 0.43 wt. %. Sample reproducibility of duplicates for C was ± 0.33 wt. % (n = 40). The average N content for NIST 1547 (Peach Leaves) was 2.81 ± 0.08 wt. % (n = 50), which compares well with its accepted value of 2.94 wt. %. Sample reproducibility of duplicates for N was ± 0.04 wt. % (n =39).

1. **Results of soil analysis**

All topsoils, except for S13-9, are dominated by silt (avg. 48.9 ± 16.5 wt. %, all ± errors reported hereafter are one standard deviation (SD)). Soil S13-9 contains > 80 wt. % sand. Mean clay concentration is 18.1 ± 7.8 wt. %. For all samples, there is a strong correlation between OC content from EA measurements and OM content as determined by loss-on-ignition (r = 0.979, *p* < 0.001). X-ray powder diffraction results indicate the presence of calcite in most samples (Table B in S1 Text), which is consistent with the average difference between TC of 2.6 ± 1.2 wt. % and OC of 1.8 ± 1.1 wt. % measured for these soils. The near neutral pH (avg. 7.8 ± 0.3) measured for all samples is also consistent with these observations. The atomic OC/TN ratio of the soils varies from 10.5 to 18.6.

**Table A. Soil characteristics.**

| **Site ID** | **Depth** | **pH** | **Texture** | **Sand** | **Silt** | **Clay** | **OM^a^** | **OC^b^** | **TC^c^** | **TN^d^** | **Atomic OC/TN** |
| --- | --- | --- | --- | --- | --- | --- | --- | --- | --- | --- | --- |
|  | **Cm** |  |  | **wt. %** | | | | | | |  |
| **S13-6** | 0-20 | 7.57 | SiL^e^ | 29.6 | 57.6 | 12.8 | 7.5 | 2.8 | 3.0 | 0.2 | 17.9 |
| **S13-6** | 20-30 | 7.75 | L^f^ | 41.6 | 43.6 | 14.8 | 6.1 | 2.2 | 2.6 | 0.1 | 18.6 |
| **S13-6** | 30-40 | 8.05 | L | 39.6 | 43.6 | 16.8 | 5.7 | 2.1 | 2.9 | 0.2 | 16.2 |
| **S13-6** | 40-45 | 8.18 | L | 39.6 | 39.6 | 20.8 | 6.1 | 2.1 | 3.0 | 0.2 | 15.4 |
| **S13-6** | 45-60 | 8.13 | L | 43.6 | 39.6 | 16.8 | 4.4 | 1.6 | 2.7 | 0.1 | 14.6 |
| **S13-6** | 60-70 | 8.38 | L | 43.6 | 40.0 | 16.4 | 2.9 | 0.5 | 2.2 | 0.1 | 12.9 |
| **S13-7** | 0-10 | 7.54 | SiL | 27.6 | 55.8 | 16.6 | 9.2 | 3.7 | 4.1 | 0.4 | 12.5 |
| **S13-8-1** | 0-10 | 7.73 | SiL | 21.6 | 56.0 | 22.4 | 8.0 | 2.5 | 4.7 | 0.3 | 10.9 |
| **S13-8-1** | 10-20 | 7.99 | SiL | 27.2 | 58.4 | 14.4 | 3.8 | 1.1 | 1.1 | 0.1 | 12.9 |
| **S13-8-1** | 20-60 | 7.72 | SiL | 19.6 | 64.0 | 16.4 | 3.4 | 0.8 | 0.8 | 0.1 | 12.8 |
| **S13-8-2** | 0-10 | 7.52 | SiL | 21.6 | 60.0 | 18.4 | 3.3 | 0.7 | 0.7 | 0.1 | 13.1 |
| **S13-9** | 0-10 | 7.71 | S | 81.6 | 10.0 | 8.4 | 2.1 | 0.5 | 1.8 | 0.1 | 12.6 |
| **S13-10-1** | 0-10 | 7.55 | L | 35.6 | 48.0 | 16.4 | 6.9 | 2.8 | 2.9 | 0.2 | 13.6 |
| **S13-10-2** | 0-10 | 7.25 | L | 37.6 | 46.0 | 16.4 | 8.5 | 3.4 | 4.0 | 0.4 | 10.5 |
| **Slims River** | 0-10 | 7.94 | SiCL^g^ | 7.6 | 57.6 | 34.8 | 2.8 | 0.2 | 3.0 | 0.0 | 16.1 |

**^a^: Organic Matter; ^b^: Organic Carbon; ^c^: Total Carbon; ^d^: Total Nitrogen; ^e^: SiL: Silty Loam; ^f^: L: Loam**

**^g^: SiCL: Silty Clay Loam**

**Table B. Soil mineralogy.**

| **Mineral/ d-space (nm)** | **Quartz** | **Alkali feldspar** | **Calcite** | **0.71^a^** | **Amphibole** | **1.01^b^** | **Dolomite** |
| --- | --- | --- | --- | --- | --- | --- | --- |
| **Site ID** | **wt. %** | | | | | | |
| **S13-6 (0-20)** | 69 | 7 | < 5 | 9 | 6 | 6 | < 5 |
| **S13-6 (20-30)** | 70 | 9 | 7 | 7 | 5 | < 5 | < 5 |
| **S13-6 (30-40)** | 60 | 9 | 8 | 5 | < 5 | < 5 | < 5 |
| **S13-6 (40-45)** | 60 | 14 | 9 | 9 | < 5 | < 5 | < 5 |
| **S13-6 (45-60)** | 64 | 17 | 6 | 8 | < 5 | < 5 | < 5 |
| **S13-6 (60-70)** | 68 | 16 | 12 | < 5 | < 5 | < 5 | < 5 |
| **S13-7 (0-10)** | 73 | 19 | < 5 | 6 | < 5 | < 5 | < 5 |
| **S13-8-1 (0-10)** | 41 | 12 | 21 | < 5 | 5 | < 5 | 18 |
| **S13-8-1 (10-20)** | 81 | 15 | < 5 | < 5 | < 5 | < 5 | < 5 |
| **S13-8-1 (20-60)** | 79 | 14 | < 5 | 6 | < 5 | < 5 | < 5 |
| **S13-8-2 (0-10)** | 80 | 14 | < 5 | 6 | < 5 | < 5 | < 5 |
| **S13-9 (0-10)** | 80 | 8 | 6 | < 5 | < 5 | < 5 | < 5 |
| **S13-10-1 (0-10)** | 79 | 39 | < 5 | 8 | < 5 | < 5 | < 5 |
| **S13-10-2 (0-10)** | 80 | 13 | < 5 | 8 | < 5 | < 5 | < 5 |
| **Slims River** | 43 | 8 | 23 | 17 | - | 9 | < 5 |

**^a^ Septechlorite/Kaolinite**

**^b^Mica/Illite**

**References:**

1. Laxton NF, Burn CR, Smith CAS. Productivity of loessal grasslands in the Kluane Lake region, Yukon Territory, and the Beringian “Production Paradox”. Arctic. 1996;49:129-140.

2. Bouyoucos GJ. Hydrometer method improved for making particle size analyses of soils. Agron J. 1962;54:464-465.

3. Kettler TA, Doran JW, Gilbert TL. Simplified method for soil particle-size determination to accompany soil-quality analyses. Soil Sci Soc Am J. 2001;65:849-852.

4. Heiri O, Lotter AF, Lemcke G. Loss on ignition as a method for estimating organic and carbonate content in sediments: reproducibility and comparability of results. J Paleolimnol. 2001;25:101-110.

5. Sheldrick B. Analytical Methods Manual 1984. Land Resource Research Institute Ottawa, Ontario, Canada; 1984.
