# Supplementary material for: Nitrogen and carbon isotopic dynamics of subarctic soils and plants in southern Yukon Territory and its implications for paleoecological and paleodietary studies
Source: PLoS One. 2017 Aug 16;12(8):e0183016. doi: 10.1371/journal.pone.0183016 (PMC5559067; doi:10.1371/journal.pone.0183016)
Supplement: S1 Table — (DOCX) [file pone.0183016.s002.docx]

**S1 Table: δ^13^C and *δ*^15^N of all plant parts.**

| **Taxonomic** |  |  | ***δ*^13^C (‰, VPDB)** | | | | | | | | | ***δ*^15^N (‰, AIR)** | | | | | | | | | |
| --- | --- | --- | --- | --- | --- | --- | --- | --- | --- | --- | --- | --- | --- | --- | --- | --- | --- | --- | --- | --- | --- |
| **Name** | **Site ID** | **Type** | **FR** | | **RC** | **S** | | **L** | | **I** | | **FR** | | **RC** | | **S** | | **L** | | **I** | |
| **2012** | | | | | | | | | | | | | | | | | | | | | |
| *P. glauca* | S12-1 | G | | *‒* | ‒29.3 | | ‒30.0 | | ‒30.0 | | ‒30.7 | | *‒* | | ‒0.3 | ‒2.7 | ‒1.3 | | ‒1.8 | |  |
| *F. altaica* | S12‒1 | G | | *‒* | ‒27.0 | | **‒26.7** | | **‒29.4** | | ‒28.8 | | *‒* | | +0.6 | +0.1 | +1.7 | | ‒1.2 | |  |
| *P. glauca* | S12‒2 | G | | *‒* | ‒27.1 | | ‒27.1 | | ‒27.8 | | ‒28.4 | | *‒* | | ‒1.0 | +1.2 | **+0.3** | | ‒0.6 | |  |
| *P. glauca* | S12‒2 | G | | ‒25.0 | ‒26.9 | | ‒27.3 | | ‒27.5 | | **‒29.4** | | +2.1 | | +2.3 | +1.2 | 1.9 | | ‒1.5 | |  |
| *P. glauca* | S12‒2 | G | | ‒26.1 | ‒26.6 | | ‒27.9 | | ‒27.6 | | ‒28.8 | | ‒0.3 | | 1.1 | ‒0.2 | ‒1.1 | | ‒1.0 | |  |
| *C. purpurascens* | S12‒2 | G | | **‒26.4** | **‒26.3** | | ‒26.0 | | ‒26.1 | | ‒27.1 | | ‒0.3 | | ‒1.0 | ‒2.1 | ‒0.9 | | ‒1.9 | |  |
| *P. gormanii* | S12‒2 | F | | *‒* | ‒26.3 | | ‒27.3 | | **‒27.6** | | ‒24.2 | | *‒* | | ‒1.0 | ‒0.9 | 2.6 | | +1.3 | |  |
| *L. lewisii* | S12‒2 | SS | | ‒26.9 | ‒26.8 | | ‒27.8 | | ‒26.5 | | *‒* | | +1.0 | | **+0.8** | +0.9 | +3.0 | | *‒* | |  |
| *L. lewisii* | S12‒2 | SS | | ‒24.0 | ‒25.2 | | ‒25.8 | | ‒25.4 | | ‒25.8 | | +1.6 | | +1.3 | +0.9 | **+3.0** | | +0.2 | |  |
| *E. trachycaulus* | S12‒3 | G | | ‒27.9 | ‒27.3 | | ‒26.7 | | ‒28.6 | | ‒27.9 | | +0.4 | | +0.7 | **‒0.1** | 0.4 | | ‒1.4 | |  |
| *E. trachycaulus* | S12‒3 | G | | ‒28.1 | ‒27.3 | | **‒26.9** | | ‒27.3 | | ‒29.1 | | 0.0 | | ‒1.3 | **+5.9** | ‒0.3 | | 0.5 | |  |
| *F. altaica* | S12‒3 | G | | ‒27.5 | ‒27.8 | | ‒27.8 | | ‒27.3 | | ‒29.2 | | +0.3 | | ‒0.5 | ‒3.4 | ‒1.5 | | ‒2.1 | |  |
| *P. glauca* | S12‒3 | G | | ‒27.7 | ‒27.3 | | ‒27.1 | | ‒27.1 | | ‒27.9 | | 0.0 | | ‒1.2 | ‒2.3 | ‒1.7 | | +0.2 | |  |
| *P. glauca* | S12‒3 | G | | **‒27.5** | ‒27.6 | | ‒27.1 | | ‒27.9 | | **‒28.2** | | **‒0.3** | | ‒1.7 | ‒2.2 | ‒1.2 | | ‒1.4 | |  |
| *F. altaica* | S12‒4 | G | | ‒28.8 | ‒28.9 | | ‒29.3 | | ‒28.6 | | ‒30.0 | | +0.4 | | +0.9 | ‒1.9 | ‒0.6 | | **‒1.1** | |  |
| *E. trachycaulus* | S12‒4 | G | | ‒28.6 | ‒29.1 | | **‒29.0** | | **‒29.4** | | ‒28.5 | | +3.3 | | +4.9 | +3.3 | +3.3 | | +3.2 | |  |
| *E. trachycaulus* | S12‒4 | G | | ‒28.74 | ‒28.95 | | **‒29.47** | | ‒29.68 | | ‒28.21 | | ‒1.2 | | ‒0.9 | +0.9 | +0.1 | | +0.8 | |  |
| *E. trachycaulus* | S12‒5 | G | | ‒28.2 | ‒28.1 | | ‒26.5 | | ‒28.2 | | ‒27.1 | | 0.0 | | +6.7 | +0.1 | **+0.8** | | ‒1.3 | |  |
|  | |  | |  |  | |  | |  | |  | |  | |  |  |  | |  | |  |
| **S1 Table. Cont’d.** | |  | |  |  | |  | |  | |  | |  | |  |  |  | |  | |  |
| **Taxonomic** |  |  | | ***δ*^13^C (‰, VPDB)** | | | | | | | | ***δ*^15^N (‰, AIR)** | | | | | | | | | |
| **Name** | **Site ID** | **Type** | | **FR** | **RC** | | **S** | | **L** | | **I** | | **FR** | | **RC** | **S** | **L** | | **I** | |  |
| *E. trachycaulus* | S12‒5 | G | | ‒27.6 | ‒28.4 | | ‒28.8 | | ‒29.3 | | ‒27.5 | | +0.5 | | +2.7 | +0.6 | +0.9 | | +1.2 | |  |
| *E. trachycaulus* | S12‒5 | G | | ‒28.1 | **‒28.1** | | ‒27.3 | | ‒29.4 | | **‒27.3** | | ‒11.1 | | ‒3.8 | ‒7.0 | ‒4.3 | | ‒0.6 | |  |
| *E. trachycaulus* | S12‒5 | G | | ‒28.4 | ‒28.1 | | ‒27.6 | | ‒28.6 | | ‒25.0 | | **‒0.3** | | +4.1 | ‒0.6 | +2.2 | | +0.9 | |  |
| *C. purpurascens* | S12‒5 | G | | ‒26.5 | ‒26.2 | | ‒25.1 | | ‒27.3 | | ‒26.7 | | ‒1.3 | | +2.1 | +0.6 | +1.1 | | +2.7 | |  |
| *P. glauca* | S12‒5 | G | | **‒28.3** | ‒28.7 | | ‒28.6 | | ‒28.9 | | ‒30.0 | | **+2.0** | | +1.7 | +0.2 | +1.8 | | +0.1 | |  |
| *P. glauca* | S12‒5 | G | | ‒28.3 | **‒28.4** | | ‒28.6 | | ‒29.3 | | ‒30.3 | | +0.7 | | **+1.2** | +0.2 | +0.9 | | +0.8 | |  |
| *E. trachycaulus* | S12‒6 | G | | ‒27.1 | ‒27.2 | | ‒27.4 | | ‒26.0 | | **‒27.5** | | ‒1.7 | | +0.9 | +0.2 | +0.3 | | +0.1 | |  |
| *E. trachycaulus* | S12‒6 | G | | ‒26.5 | ‒26.3 | | ‒25.6 | | ‒27.9 | | ‒26.2 | | +2.2 | | +2.4 | +0.5 | +2.3 | | ‒0.2 | |  |
| *E. trachycaulus* | S12‒6 | G | | ‒27.6 | ‒27.6 | | ‒27.8 | | ‒27.8 | | ‒27.5 | | +0.3 | | +1.9 | +0.5 | +0.6 | | +1.2 | |  |
| *E. trachycaulus* | S12‒6 | G | | ‒27.3 | ‒27.3 | | ‒27.9 | | ‒28.3 | | ‒27.5 | | +1.9 | | **+2.1** | +0.1 | +2.7 | | **+0.5** | |  |
| *C. purpurascens* | S12‒6 | G | | ‒25.4 | ‒25.9 | | ‒25.1 | | ‒25.4 | | ‒26.0 | | ‒1.0 | | ‒1.5 | ‒1.3 | ‒1.3 | | ‒3.2 | |  |
| *P. glauca* | S12‒6 | G | | ‒26.2 | ‒26.3 | | ‒27.3 | | ‒27.3 | | ‒27.9 | | ‒1.8 | | ‒1.4 | ‒2.0 | ‒1.3 | | ‒1.2 | |  |
| *P. glauca* | S12‒6 | G | | ‒25.7 | ‒26.1 | | ‒25.2 | | ‒26.4 | | ‒25.7 | | ‒1.3 | | ‒2.0 | ‒2.0 | ‒1.0 | | ‒0.4 | |  |
| **2013** | | | | | | | | | | | | | | | | | | | | |  |
| *E. trachycaulus* | S13‒3 | G | | ‒28.3 | ‒28.6 | | ‒28.7 | | ‒29.4 | | ‒27.0 | | ‒1.4 | | ‒2.4 | ‒2.0 | ‒2.3 | | ‒2.1 | |  |
| *E. spicatus* | S13‒7 | G | | ‒27.1 | ‒28.7 | | ‒25.0 | | ‒28.3 | | ‒26.7 | | ‒1.4 | | ‒1.9 | ‒1.5 | ‒1.4 | | ‒2.1 | |  |
| *C. filifolia* | S13‒8 | SG | | ‒28.5 | ‒26.9 | | ‒28.5 | | ‒28.2 | | ‒27.7 | | ‒1.2 | | ‒2.0 | ‒0.7 | 0.0 | | ‒0.9 | |  |
| *E. trachycaulus* | S13‒3 | G | | ‒28.3 | ‒28.7 | | ‒28.2 | | ‒29.1 | | ‒28.4 | | ‒1.9 | | ‒2.5 | ‒2.8 | ‒2.1 | | ‒1.9 | |  |
| *C. purpurascens* | S13‒2 | G | | ‒27.7 | ‒28.2 | | ‒26.5 | | ‒27.9 | | ‒27.5 | | 0.2 | | ‒0.5 | ‒1.0 | ‒0.3 | | ‒0.7 | |  |
| **S1 Table. Cont’d.** | |  | |  |  | |  | |  | |  | |  | |  |  |  | |  | |  |
| **Taxonomic** |  |  | | ***δ*^13^C (‰, VPDB)** | | | | | | | | ***δ*^15^N (‰, AIR)** | | | | | | | | | |
| **Name** | **Site ID** | **Type** | | **FR** | **RC** | | **S** | | **L** | | **I** | | **FR** | | **RC** | **S** | **L** | | **I** | |  |
| *B.pumpellianus* | S13‒3 | G | | ‒26.6 | ‒25.7 | | **‒26.4** | | ‒27.6 | | ‒27.3 | | 0.2 | | ‒0.8 | ‒1.0 | ‒0.5 | | ‒0.6 | |  |
| *E. spicatus* | S13‒7 | G | | ‒27.3 | ‒28.1 | | ‒25.4 | | ‒27.1 | | ‒25.7 | | ‒1.2 | | ‒2.3 | ‒3.1 | ‒1.9 | | ‒1.1 | |  |
| *C. purpurascens* | S13‒3 | G | | **‒26.5** | **‒27.0** | | ‒26.2 | | ‒27.4 | | **‒27.2** | | ‒1.5 | | **‒1.9** | ‒3.1 | ‒2.3 | | **‒1.6** | |  |
| *A. frigida* | S13‒2 | SS | | **‒28.3** | **‒29.3** | | **‒29.3** | | ‒30.9 | | **‒27.2** | | +5.5 | | **+15.3** | +25.4 | +23.4 | | +21.2 | |  |
| *A. frigida* | S13‒2 | SS | | ‒28.9 | ‒28.8 | | **‒29.2** | | ‒30.3 | | ‒28.8 | | +12.8 | | +6.8 | +22.9 | +18.7 | | **+27.4** | |  |
| *E. trachycaulus* | S13‒3 | G | | ‒27.8 | ‒27.9 | | ‒27.2 | | ‒28.9 | | ‒26.5 | | ‒1.3 | | ‒1.9 | ‒2.4 | **‒2.2** | | ‒1.4 | |  |
| *B.pumpellianus* | S13‒6 | G | | ‒27.0 | ‒28.2 | | ‒26.7 | | ‒27.5 | | ‒27.8 | | +3.1 | | +2.6 | +2.1 | +2.9 | | +2.3 | |  |
| *E.trachycaulust* | S13‒6 | G | | ‒27.5 | ‒28.0 | | ‒27.6 | | ‒28.1 | | ‒27.2 | | +3.1 | | +2.4 | +2.5 | +3.5 | | +2.9 | |  |
| *C. purpurascens* | S13‒6 | G | | ‒25.6 | ‒25.9 | | ‒24.7 | | ‒25.5 | | ‒25.6 | | +1.4 | | +1.0 | ‒0.8 | +1.4 | | +0.9 | |  |
| *P. glauca* | S13‒5 | G | | ‒25.8 | ‒26.5 | | ‒24.4 | | ‒25.8 | | ‒25.4 | | +5.4 | | +4.1 | 3.9 | +1.8 | | +2.3 | |  |
| *P. canescens* | S13‒2 | F | | ‒27.7 | ‒27.6 | | ‒27.0 | | ‒28.7 | | ‒26.5 | | ‒0.8 | | ‒1.0 | **‒0.9** | **‒0.9** | | 0.0 | |  |
| *C. purpurascens* | S13‒2 | G | | ‒26.9 | ‒27.3 | | ‒26.9 | | ‒26.9 | | ‒27.5 | | +0.6 | | ‒0.4 | 0.0 | 0.0 | | ‒0.5 | |  |
| *L.ramosissimum* | S13‒8 | F | | **‒26.9** | ‒ | | ‒26.0 | | ‒25.9 | | ‒ | | +3.7 | | ‒ | +4.2 | +7.0 | | ‒ | |  |
| *B. glandulosa* | S13‒11 | S | | ‒ | ‒ | | ‒30.5 | | ‒30.9 | | ‒ | | ‒ | | ‒ | ‒5.0 | ‒5.1 | | ‒ | |  |
| *P. glauca* | S13‒4 | G | | ‒25.8 | ‒26.4 | | ‒24.4 | | ‒26.6 | | **‒24.8** | | +2.2 | | +1.3 | +1.9 | +2.1 | | **+1.9** | |  |
| *E. trachycaulus* | S13‒3 | G | | ‒27.8 | ‒29.0 | | ‒29.1 | | ‒29.9 | | ‒28.1 | | ‒1.3 | | ‒1.7 | ‒0.7 | ‒1.1 | | ‒0.2 | |  |
| *E. trachycaulus* | S13‒4 | G | | ‒ | ‒ | | ‒23.5 | | ‒26.5 | | ‒24.3 | | ‒ | | ‒ | +3.3 | +2.8 | | +4.0 | |  |
| *E. trachycaulus* | S13‒6 | G | | ‒27.1 | ‒27.5 | | ‒24.7 | | ‒27.7 | | ‒25.8 | | +0.5 | | ‒1.0 | ‒1.0 | ‒0.5 | | 0.0 | |  |
| *E. spicatus* | S13‒7 | G | | ‒27.3 | ‒26.0 | | ‒24.6 | | ‒28.4 | | ‒25.5 | | ‒0.3 | | ‒2.1 | ‒3.1 | ‒2.8 | | ‒1.1 | |  |
| **S1 Table. Cont’d.** | |  | |  |  | |  | |  | |  | |  | |  |  |  | |  | |  |
| **Taxonomic** |  |  | | ***δ*^13^C (‰, VPDB)** | | | | | | | | ***δ*^15^N (‰, AIR)** | | | | | | | | | |
| **Name** | **Site ID** | **Type** | | **FR** | **RC** | | **S** | | **L** | | **I** | | **FR** | | **RC** | **S** | **L** | | **I** | |  |
| *F. altaica* | S13‒11 | G | | ‒27.7 | ‒27.5 | | ‒27.5 | | ‒27.6 | | ‒27.8 | | ‒3.9 | | ‒6.3 | ‒6.2 | ‒7.2 | | ‒6.2 | |  |
| *A. frigida* | S13‒4 | SS | | **‒27.7** | ‒27.9 | | ‒26.1 | | ‒29.7 | | ‒25.4 | | +1.3 | | +3.6 | +1.7 | +4.8 | | +3.6 | |  |
| *C. filifolia* | S13‒6 | SG | | ‒26.3 | ‒25.6 | | ‒27.9 | | ‒27.7 | | ‒27.5 | | +0.2 | | +0.3 | +1.7 | 1.3 | | **+3.1** | |  |
| *E. trachycaulus* | S13‒3 | G | | ‒27.1 | ‒27.2 | | ‒26.8 | | ‒28.2 | | ‒24.6 | | +3.2 | | **+2.6** | +0.6 | +1.1 | | +3.8 | |  |
| *B.pumpellianus* | S13‒3 | G | | ‒26.8 | ‒26.2 | | ‒25.5 | | ‒28.4 | | ‒27.1 | | ‒1.2 | | ‒0.8 | ‒1.6 | ‒1.5 | | ‒1.8 | |  |
| *P. canescens* | S13‒2 | F | | ‒27.2 | ‒26.2 | | ‒24.7 | | ‒28.6 | | ‒27.4 | | **+0.1** | | ‒0.5 | +5.7 | +0.3 | | **+0.7** | |  |
| *B.pumpellianus* | S13‒3 | G | | ‒26.2 | ‒26.8 | | ‒25.7 | | ‒27.7 | | ‒27.4 | | ‒0.7 | | ‒1.0 | ‒1.2 | ‒0.1 | | ‒0.8 | |  |
| *A. frigida* | S13‒6 | SS | | ‒28.3 | ‒28.2 | | ‒28.2 | | **‒27.3** | | **‒26.5** | | +1.0 | | +1.1 | +1.4 | +2.1 | | +3.2 | |  |
| *L. lewisii* | S13‒10 | SS | | ‒26.0 | ‒27.8 | | ‒27.4 | | ‒27.9 | | ‒27.6 | | +2.7 | | +2.7 | +0.8 | **+2.9** | | +3.0 | |  |
| *C. purpurascens* | S13‒2 | G | | ‒26.9 | ‒27.4 | | ‒26.9 | | ‒27.3 | | ‒27.9 | | +1.1 | | +0.2 | +0.2 | 0.1 | | +1.0 | |  |
| *A. frigida* | S13‒7 | SS | | ‒28.8 | ‒29.8 | | ‒29.2 | | ‒28.2 | | **‒29.3** | | ‒1.2 | | ‒0.4 | ‒2.4 | ‒0.6 | | ‒1.9 | |  |
| *P. glauca* | S13‒6 | G | | ‒ | ‒ | | ‒27.3 | | ‒28.2 | | ‒27.4 | | ‒ | | ‒ | ‒2.5 | ‒2.6 | | ‒1.7 | |  |
| *C. purpurascens* | S13‒3 | G | | ‒26.5 | ‒24.0 | | ‒24.8 | | ‒25.9 | | ‒26.3 | | ‒1.3 | | ‒1.8 | ‒3.2 | ‒2.4 | | ‒3.3 | |  |
| *F. altaica* | S13‒11 | G | | ‒ | ‒ | | **‒28.9** | | ‒30.5 | | ‒29.7 | | ‒ | | ‒ | ‒5.6 | ‒4.9 | | ‒4.4 | |  |
| *E. spicatus* | S13‒7 | G | | ‒28.5 | ‒28.9 | | ‒26.9 | | ‒28.4 | | ‒28.4 | | ‒1.1 | | ‒2.5 | ‒3.3 | ‒2.2 | | 0.0 | |  |
| *P. glauca* | S13‒4 | G | | ‒25.9 | **‒25.5** | | ‒24.3 | | ‒25.5 | | ‒26.6 | | +0.3 | | +1.4 | +0.6 | +1.4 | | +1.3 | |  |
| *A. frigida* | S13‒6 | SS | | ‒28.1 | ‒ | | ‒29.2 | | ‒28.5 | | ‒ | | 0.0 | | ‒ | +1.0 | +2.9 | | ‒ | |  |
| *R. idaeus* | S13‒13 | S | | ‒ | ‒ | | ‒27.4 | | **‒27.9** | | ‒ | | ‒ | | ‒ | ‒3.5 | ‒3.9 | | ‒ | |  |
| *B.pumpellianus* | S13‒3 | G | | ‒26.6 | ‒26.4 | | ‒26.9 | | ‒28.1 | | ‒27.5 | | ‒0.2 | | ‒0.9 | ‒2.2 | ‒1.3 | | ‒1.7 | |  |
| **S1 Table. Cont’d.** | |  | |  |  | |  | |  | |  | |  | |  |  |  | |  | |  |
| **Taxonomic** |  |  | | ***δ*^13^C (‰, VPDB)** | | | | | | | | ***δ*^15^N (‰, AIR)** | | | | | | | | | |
| **Name** | **Site ID** | **Type** | | **FR** | **RC** | | **S** | | **L** | | **I** | | **FR** | | **RC** | **S** | **L** | | **I** | |  |
| *E. spicatus* | S13‒7 | G | | ‒27.6 | ‒28.0 | | ‒26.9 | | ‒28.3 | | ‒26.3 | | ‒1.3 | | ‒2.2 | ‒4.2 | ‒2.7 | | ‒2.1 | |  |
| *C. purpurascens* | S13‒2 | G | | ‒27.9 | ‒26.9 | | **‒26.5** | | ‒27.8 | | ‒26.6 | | ‒2.5 | | **‒2.5** | ‒4.7 | ‒3.6 | | ‒4.4 | |  |
| *R. idaeus* | S13‒14 | S | | ‒ | ‒ | | ‒32.2 | | ‒30.6 | | ‒ | | ‒ | | ‒ | ‒1.2 | ‒0.9 | | ‒ | |  |
| *F. altaica* | S13‒11 | G | | ‒28.6 | ‒28.8 | | ‒27.8 | | **‒27.0** | | ‒28.1 | | ‒3.9 | | ‒4.4 | ‒9.5 | ‒8.3 | | ‒8.0 | |  |
| *S. arctica* | S13‒15 | S | | ‒ | ‒ | | **‒26.6** | | ‒27.5 | | ‒27.3 | | ‒ | | ‒ | **‒4.0** | ‒3.5 | | ‒3.2 | |  |

***A. frigidia*: *Artemisia frigida*;**

***B. glandulosa: Betula glandulosa*;**

***B. pumpellianus: Bromus pumpellianus*;**

***C. filifolia*: *Carex filifolia*;**

***C. purpurascens: Calamograstis purpurascens*;**

***E. spicatus*: *Elymus spicatus*;**

***E. trachycaulus: Elymus trachycaulus*;**

***F. altaica*: *Festuca altaica*;**

***L. lewissii*: *Linum Lewisii*;**

***L. ramosissimum: Lepidium ramosissimum*;**

***P. canescens: Plantago canescens*;**

***P. glauca*: *Poa glauca*;**

***P. gormanii: Penstemon gormanii*;**

***R. idaeus*: *Rubus idaeus*;**

***S. arctica*: *Salix arctica*;**

**FR: Fine root; RC: Root crown; S: Stem; L: Leaf; I: Inflorescence.**

**G: Grass; S: Shrub; SS: Subshrub; SG: Sedge; F: Forb.**

**Boldface denotes average of duplicates.**
