# Supplementary material for: Nitrogen and carbon isotopic dynamics of subarctic soils and plants in southern Yukon Territory and its implications for paleoecological and paleodietary studies
Source: PLoS One. 2017 Aug 16;12(8):e0183016. doi: 10.1371/journal.pone.0183016 (PMC5559067; doi:10.1371/journal.pone.0183016)
Supplement: S2 Table — (DOCX) [file pone.0183016.s003.docx]

**S2 Table:** **Carbon and nitrogen contents and foliar atomic C/N of all plants.**

| **Taxonomic** |  |  | | **C (wt. %)** | | | | | | | | | **N (wt. %)** | | | | | | | | | | **Atomic C/N** |
| --- | --- | --- | --- | --- | --- | --- | --- | --- | --- | --- | --- | --- | --- | --- | --- | --- | --- | --- | --- | --- | --- | --- | --- |
| **Name** | **Site ID** | **Type** | | **FR** | **RC** | **S** | | **L** | | **I** | | **FR** | | **RC** | | **S** | | **L** | | **I** | | **Foliar** | |
| **2012** | | | | | | | | | | | | | | | | | | | | | |  | |
| *P. glauca* | S12‒1 | G | *‒* | | 43.3 | | 42.9 | | 41.4 | | 42.1 | | *‒* | | 1.4 | | 0.3 | | 0.6 | | 1.0 | | 80.5 |
| *F. altaica* | S12‒1 | G | *‒* | | **42.8** | | **43.9** | | **41.5** | | 43.3 | | *‒* | | 1.5 | | 0.1 | | 1.1 | | 0.4 | | 44.0 |
| *P. glauca* | S12‒2 | G | *‒* | | 41.2 | | 41.8 | | 41.0 | | 38.6 | | *‒* | | 1.9 | | 1.0 | | 0.5 | | 0.7 | | 95.6 |
| *P. glauca* | S12‒2 | G | 45.9 | | **44.0** | | 44.2 | | 41.9 | | **41.8** | | 1.0 | | **1.9** | | 0.9 | | 1.6 | | 0.4 | | 30.5 |
| *P. glauca* | S12‒2 | G | 44.7 | | 40.9 | | 42.4 | | 40.6 | | 41.7 | | 0.9 | | 1.5 | | 0.5 | | 0.7 | | 0.7 | | 67.6 |
| *C. purpurascens* | S12‒2 | G | 43.6 | | **40.5** | | 44.1 | | 40.3 | | 42.3 | | 0.5 | | 0.9 | | 0.3 | | 0.3 | | 1.3 | | 156.7 |
| *P. gormanii* | S12‒2 | F | *‒* | | 43.3 | | 45.1 | | **45.8** | | 45.4 | | *‒* | | 1.2 | | 0.4 | | 1.9 | | 0.9 | | 28.1 |
| *L. lewisii* | S12‒2 | SS | 47.2 | | 46.8 | | 46.0 | | 45.5 | | *‒* | | 1.7 | | 2.0 | | 1.3 | | 4.0 | | *‒* | | 13.3 |
| *L. lewisii* | S12‒2 | SS | 46.4 | | 46.0 | | 45.2 | | 44.4 | | 44.4 | | 1.5 | | 1.7 | | 0.9 | | **3.5** | | 1.0 | | 14.8 |
| *E. trachycaulus* | S12‒3 | G | 41.7 | | 41.3 | | 44.2 | | 40.0 | | 43.4 | | 0.8 | | 1.2 | | 0.2 | | 0.8 | | 0.5 | | 58.3 |
| *E. trachycaulus* | S12‒3 | G | **44.9** | | 41.8 | | **45.2** | | 43.5 | | 43.4 | | **0.7** | | 1.1 | | **0.2** | | 0.5 | | 0.7 | | 101.5 |
| *F. altaica* | S12‒3 | G | 39.9 | | 33.0 | | 43.4 | | 37.5 | | 41.4 | | 0.5 | | 0.9 | | 0.1 | | 0.5 | | 0.4 | | 87.5 |
| *P. glauca* | S12‒3 | G | 43.7 | | 41.8 | | 42.4 | | 40.3 | | **39.7** | | 0.6 | | 1.4 | | 0.4 | | 0.5 | | 0.7 | | 94.0 |
| *P. glauca* | S12‒3 | G | 41.2 | | 41.0 | | 46.1 | | 40.8 | | 43.6 | | **0.8** | | 1.1 | | 0.2 | | 0.9 | | 0.3 | | 52.9 |
| *F. altaica* | S12‒4 | G | 38.4 | | 43.0 | | 45.3 | | 41.0 | | 42.4 | | 0.9 | | 1.3 | | 0.2 | | 0.5 | | 0.5 | | 95.6 |
| *E. trachycaulus* | S12‒4 | G | **43.9** | | 44.6 | | **44.7** | | **41.6** | | 43.7 | | **0.8** | | 1.2 | | 0.2 | | 0.5 | | 0.9 | | 97.0 |
| *E. trachycaulus* | S12‒4 | G | 43.5 | | 43.1 | | **44.6** | | 41.6 | | 44.2 | | 0.9 | | 1.2 | | 0.2 | | 0.4 | | 1.4 | | 121.3 |
|  |  |  |  | |  | |  | |  | |  | |  | |  | |  | |  | |  | |  |
|  |  |  |  | |  | |  | |  | |  | |  | |  | |  | |  | |  | |  |
| **S2 Table. Cont’d.** | |  |  | |  | |  | |  | |  | |  | |  | |  | |  | |  | |  |
| **Taxonomic** |  |  | **C (wt. %)** | | | | | | | | | | **N (wt. %)** | | | | | | | | | | **Atomic C/N** |
| **Name** | **Site ID** | **Type** | **FR** | | **RC** | | **S** | | **L** | | **I** | | **FR** | | **RC** | | **S** | | **L** | | **I** | | **Foliar** |
| *E. trachycaulus* | S12‒5 | G | 42.9 | | 43.4 | | 45.1 | | **41.1** | | **43.4** | | 1.2 | | 1.6 | | 0.1 | | **0.8** | | **0.4** | | 59.9 |
| *E. trachycaulus* | S12‒6 | G | 43.0 | | 42.8 | | 45.3 | | 38.7 | | 44.0 | | 1.1 | | 1.12 | | 0.1 | | 0.5 | | 0.9 | | 90.3 |
| *E. trachycaulus* | S12‒6 | G | 41.7 | | **43.4** | | 44.9 | | 40.8 | | **44.1** | | 1.1 | | 1.4 | | 0.2 | | 1.1 | | 0.5 | | 43.3 |
| *E. trachycaulus* | S12‒6 | G | 42.1 | | 40.5 | | 43.3 | | 41.4 | | 44.4 | | 1.1 | | 1.3 | | 0.2 | | 0.7 | | 0.6 | | 69.0 |
| *C. purpurascens* | S12‒6 | G | 44.1 | | 43.5 | | 43.6 | | 41.2 | | 43.3 | | 0.8 | | 1.5 | | 0.3 | | 0.5 | | 0.8 | | 96.1 |
| *P. glauca* | S12‒6 | G | 44.4 | | 43.7 | | 46.8 | | 41.1 | | 42.6 | | **0.8** | | 1.1 | | 0.1 | | 1.1 | | 0.3 | | 43.6 |
| *P. glauca* | S12‒6 | G | 42.9 | | **39.2** | | 44.3 | | 40.2 | | 41.4 | | 1.0 | | 1.3 | | 0.2 | | 0.6 | | 0.5 | | 78.1 |
| *E. trachycaulus* | S12‒7 | G | 43.0 | | 43.1 | | 43.6 | | 40.7 | | **41.2** | | 0.8 | | 1.4 | | 0.2 | | 1.1 | | 0.6 | | 43.2 |
| *E. trachycaulus* | S12‒7 | G | 43.3 | | **43.4** | | 44.6 | | 43.1 | | 43.4 | | 1.1 | | **1.5** | | 0.3 | | 1.0 | | 0.3 | | 50.3 |
| *E. trachycaulus* | S12‒7 | G | 42.4 | | 41.5 | | 44.3 | | 41.4 | | 41.4 | | 0.9 | | 1.3 | | 0.1 | | 0.7 | | 0.6 | | 69.0 |
| *E. trachycaulus* | S12‒7 | G | 42.9 | | 40.4 | | 44.2 | | 42.6 | | 43.4 | | 0.8 | | 1.4 | | 0.3 | | 0.9 | | **0.3** | | 55.2 |
| *C. purpurascens* | S12‒7 | G | 45.5 | | 43.2 | | 45.1 | | 38.6 | | 41.4 | | 0.6 | | 1.1 | | 0.3 | | 0.4 | | 0.4 | | 112.5 |
| *P. glauca* | S12‒7 | G | 44.4 | | 44.1 | | 41.5 | | 41.7 | | 42.9 | | 0.8 | | 1.4 | | 0.6 | | 0.8 | | 0.9 | | 60.8 |
| *P. glauca* | S12‒7 | G | 44.3 | | 44.2 | | 42.2 | | 41.4 | | 43.2 | | 1.09 | | **1.34** | | 0.54 | | 0.8 | | 1.07 | | 60.4 |
| **2013** | | | | | | | | | | | | | | | | | | | | | | |  |
| *E. trachycaulus* | S13‒3 | G | 38.3 | | 38.2 | | 43.4 | | 37.1 | | 42.4 | | 1.0 | | 1.4 | | 0.1 | | 1.2 | | 1.3 | | 36.1 |
| *E. spicatus* | S13‒7 | G | 35.4 | | 40.4 | | 43.7 | | 41.0 | | 42.9 | | 0.6 | | 1.1 | | 0.1 | | 1.0 | | 0.4 | | 47.8 |
| *C. filifolia* | S13‒8 | SG | 45.6 | | 46.1 | | 43.5 | | 42.9 | | 44.6 | | 0.9 | | 1.4 | | 1.3 | | 2.4 | | 1.1 | | 20.9 |
| *E. trachycaulus* | S13‒3 | G | 40.5 | | **41.7** | | 44.9 | | 38.5 | | 42.0 | | 1.1 | | **1.6** | | 0.3 | | **0.4** | | 0.5 | | 112.2 |
| **S2 Table. Cont’d.** | |  |  | |  | |  | |  | |  | |  | |  | |  | |  | |  | |  |
| **Taxonomic** |  |  | **C (wt. %)** | | | | | | | | | | **N (wt. %)** | | | | | | | | | | **Atomic C/N** |
| **Name** | **Site ID** | **Type** | **FR** | | **RC** | | **S** | | **L** | | **I** | | **FR** | | **RC** | | **S** | | **L** | | **I** | | **Foliar** |
| *C. purpurascens* | S13‒2 | G | 40.7 | | 41.9 | | 44.7 | | 39.3 | | 43.0 | | 0.6 | | 0.9 | | 0.2 | | 1.0 | | 0.5 | | 45.8 |
| *B. pumpellianus* | S13‒3 | G | 41.4 | | 42.7 | | 43.9 | | 41.0 | | 39.5 | | 0.7 | | 1.56 | | 0.5 | | 1.2 | | 0.5 | | 39.8 |
| *E. spicatus* | S13‒7 | G | 38.7 | | 42.4 | | 44.4 | | 36.6 | | 43.3 | | 0.7 | | 1.3 | | 0.2 | | 0.5 | | 0.4 | | 85.4 |
| *C. purpurascens* | S13‒3 | G | **42.1** | | **43.5** | | 45.4 | | 38.7 | | **39.7** | | 0.7 | | 1.4 | | 0.2 | | 0.5 | | 0.6 | | 90.3 |
| *A. frigida* | S13‒2 | SS | 46.4 | | 48.5 | | 45.3 | | 45.6 | | 46.7 | | 1.0 | | 1.8 | | **0.7** | | 0.6 | | 1.3 | | 88.6 |
| *A. frigida* | S13‒2 | SS | 46.2 | | 47.6 | | **45.5** | | 45.6 | | 45.7 | | 1.0 | | 1.0 | | **0.6** | | 0.9 | | **1.3** | | 59.1 |
| *E. trachycaulus* | S13‒3 | G | 43.5 | | **38.5** | | 44.3 | | 40.3 | | 43.4 | | 0.9 | | **1.5** | | 0.3 | | 1.0 | | 1.3 | | 47.0 |
| *B. pumpellianus* | S13‒6 | G | 40.3 | | 41.3 | | 42.5 | | 41.1 | | 42.6 | | 0.9 | | 1.4 | | 0.3 | | 2.3 | | 0.2 | | 20.8 |
| *E. rachycaulust* | S13‒6 | G | **40.8** | | 41.7 | | 44.8 | | 39.8 | | 43.0 | | **1.1** | | 1.2 | | 0.3 | | 1.5 | | 0.3 | | 30.9 |
| *C. purpurascens* | S13‒6 | G | 44.5 | | **45.8** | | 44.1 | | 43.2 | | 43.0 | | 0.8 | | **1.5** | | 0.3 | | 1.6 | | 1.0 | | 31.5 |
| *P. glauca* | S13‒5 | G | 43.2 | | 41.5 | | 44.5 | | 44.5 | | 44.2 | | 1.4 | | 1.4 | | 0.8 | | 1.1 | | 1.0 | | 47.2 |
| *P. canescens* | S13‒2 | F | 42.4 | | 41.6 | | 45.1 | | 39.7 | | 44.2 | | 1.2 | | 1.0 | | 0.2 | | 1.5 | | 1.2 | | 30.9 |
| *C. purpurascens* | S13‒2 | G | 41.6 | | 39.8 | | 43.5 | | 40.1 | | **42.4** | | 0.6 | | 1.1 | | 0.2 | | 1.2 | | 0.6 | | 39.0 |
| *L. ramosissimum* | S13‒8 | F | 43.5 | | ‒ | | 42.9 | | 43.7 | | ‒ | | 0.9 | | ‒ | | 2.7 | | 3.8 | | ‒ | | 13.4 |
| *B. glandulosa* | S13‒11 | S | ‒ | | ‒ | | 48.5 | | 48.0 | | ‒ | | ‒ | | ‒ | | 1.4 | | 1.6 | | ‒ | | 35.0 |
| *P. glauca* | S13‒4 | G | 41.8 | | 39.5 | | 42.6 | | 40.5 | | 37.7 | | 0.8 | | 1.1 | | 1.4 | | 1.0 | | 1.1 | | 47.2 |
| *E. trachycaulus* | S13‒3 | G | **44.4** | | **35.5** | | 45.3 | | 40.2 | | 42.6 | | **1.0** | | **1.5** | | 0.2 | | 0.7 | | 0.3 | | 67.0 |
| *E. trachycaulus* | S13‒4 | G | ‒ | | ‒ | | 43.3 | | 42.5 | | 43.5 | | ‒ | | ‒ | | 0.7 | | 0.8 | | 1.0 | | 62.0 |
| *E. trachycaulus* | S13‒6 | G | 39.0 | | 44.2 | | 45.3 | | 42.2 | | 43.4 | | 0.8 | | 1.5 | | 0.4 | | 0.8 | | 0.5 | | 61.5 |
| **S2 Table. Cont’d.** | |  |  | |  | |  | |  | |  | |  | |  | |  | |  | |  | |  |
| **Taxonomic** |  |  |  | |  | | **C (wt. %)** | |  | |  | | **N (wt. %)** | | | | | | | | | | **Atomic C/N** |
| **Name** | **Site ID** | **Type** | **FR** | | **RC** | | **S** | | **L** | | **I** | | **FR** | | **RC** | | **S** | | **L** | | **I** | | **Foliar** |
| *E. spicatus* | S13‒7 | G | 36.6 | | 39.8 | | 45.6 | | 38.7 | | 44.1 | | 0.8 | | 1.0 | | 0.2 | | 0.5 | | 0.4 | | 90.3 |
| *F. altaica* | S13‒11 | G | 42.2 | | 40.8 | | **45.3** | | 41.0 | | 39.9 | | 0.8 | | 1.6 | | 0.1 | | 1.0 | | 0.7 | | 47.8 |
| *A. frigida* | S13‒4 | SS | **41.2** | | 44.1 | | 44.9 | | 44.0 | | 46.1 | | **1.6** | | 1.6 | | 1.4 | | 3.0 | | 1.8 | | 17.1 |
| *C. filifolia* | S13‒6 | SG | 44.1 | | 47.5 | | 42.0 | | 41.9 | | 43.1 | | 0.7 | | 0.5 | | 0.7 | | 0.8 | | 1.1 | | 61.1 |
| *E. trachycaulus* | S13‒3 | G | 39.0 | | **40.2** | | 44.6 | | 42.2 | | 40.2 | | 0.9 | | 1.6 | | 0.5 | | 0.9 | | 1.3 | | 54.7 |
| *B. pumpellianus* | S13‒3 | G | 40.4 | | 39.5 | | **43.4** | | 39.2 | | 41.4 | | 1.0 | | 2.0 | | 0.4 | | 0.6 | | 0.4 | | 76.2 |
| *P. canescens* | S13‒2 | F | 42.8 | | 41.7 | | 43.8 | | 37.7 | | 43.2 | | **0.8** | | 1.2 | | **0.3** | | 1.2 | | **0.9** | | 36.6 |
| *B. pumpellianus* | S13‒3 | G | 30.5 | | 41.7 | | 43.8 | | 40.2 | | 41.8 | | 0.8 | | 1.7 | | 0.4 | | 1.7 | | 0.4 | | 27.6 |
| *A. frigida* | S13‒6 | SS | 48.4 | | 49.9 | | 45.0 | | **39.0** | | 44.5 | | 1.5 | | 1.1 | | 0.7 | | **2.1** | | 1.4 | | 21.7 |
| *L. lewisii* | S13‒10 | SS | 46.2 | | 47.1 | | 46.6 | | 43.0 | | 47.4 | | 1.2 | | 1.5 | | 0.5 | | **3.1** | | 1.4 | | 16.2 |
| *C. purpurascens* | S13‒2 | G | 43.0 | | 38.7 | | 43.4 | | 34.7 | | 42.7 | | 0.4 | | **0.9** | | 0.3 | | 0.4 | | **0.8** | | 101.2 |
| *A. frigida* | S13‒7 | SS | 41.9 | | 50.4 | | 45.4 | | 45.3 | | 45.7 | | 1.1 | | 1.2 | | 0.8 | | 2.3 | | 1.4 | | 23.0 |
| *P. glauca* | S13‒6 | G | ‒ | | ‒ | | 42.7 | | 41.1 | | 41.1 | | ‒ | | ‒ | | 1.1 | | 0.5 | | 1.6 | | 95.9 |
| *C. purpurascens* | S13‒3 | G | 42.2 | | 41.3 | | 43.7 | | 39.0 | | 40.9 | | 0.7 | | 1.4 | | 0.3 | | 0.6 | | 0.6 | | 75.8 |
| *F. altaica* | S13‒11 | G | ‒ | | ‒ | | **44.4** | | 43.3 | | 41.7 | | ‒ | | ‒ | | **0.2** | | 0.4 | | 0.5 | | 126.2 |
| *E. spicatus* | S13‒7 | G | 41.8 | | 39.9 | | 45.3 | | 40.6 | | 44.1 | | 0.9 | | 1.2 | | 0.9 | | 0.4 | | 0.6 | | 118.4 |
| *P. glauca* | S13‒4 | G | 42.6 | | 41.4 | | 43.0 | | 40.1 | | 36.4 | | 1.1 | | 2.0 | | 1.2 | | 1.3 | | **1.1** | | 36.0 |
| *A. frigida* | S13‒6 | SS | 49.5 | | ‒ | | 46.8 | | **42.7** | | ‒ | | 1.0 | | ‒ | | 1.0 | | 2.0 | | ‒ | | 24.9 |
| *R. idaeus* | S13‒13 | S | ‒ | | ‒ | | 45.4 | | **45.6** | | ‒ | | ‒ | | ‒ | | **0.8** | | 1.8 | | ‒ | | 29.5 |
| **S2 Table. Cont’d.** | |  |  | |  | |  | |  | |  | |  | |  | |  | |  | |  | |  |
| **Taxonomic** |  |  | **C (wt. %)** | | | | | | | | | | **N (wt. %)** | | | | | | | | | | **Atomic C/N** |
| **Name** | **Site ID** | **Type** | **FR** | | **RC** | | **S** | | **L** | | **I** | | **FR** | | **RC** | | **S** | | **L** | | **I** | | **Foliar** |
| *B. pumpellianus* | S13‒3 | G | 37.5 | | 39.9 | | 43.0 | | 38.4 | | 41.0 | | 0.8 | | 1.8 | | 0.3 | | 0.8 | | 0.5 | | 56.0 |
| *E. spicatus* | S13‒7 | G | 39.7 | | 40.8 | | 45.1 | | 41.8 | | 43.7 | | 1.3 | | 1.2 | | 0.2 | | 0.8 | | 0.3 | | 60.9 |
| *C. purpurascens* | S13‒2 | G | 43.6 | | 29.3 | | 44.0 | | 41.0 | | 40.6 | | 0.8 | | 0.8 | | 0.2 | | 1.0 | | 0.5 | | 47.8 |
| *R. idaeus* | S13‒14 | S | ‒ | | ‒ | | **46.3** | | 44.2 | | ‒ | | ‒ | | ‒ | | **0.6** | | 1.9 | | ‒ | | 27.1 |
| *F. altaica* | S13‒11 | G | 43.9 | | 41.6 | | 45.3 | | 41.1 | | 41.4 | | 0.7 | | 1.0 | | 0.2 | | 0.8 | | 0.6 | | 59.9 |
| *S. arctica* | S13‒15 | S | ‒ | | ‒ | | 47.7 | | **45.3** | | **45.8** | | ‒ | | ‒ | | 1.4 | | **0.9** | | **0.9** | | 58.7 |

***A. frigidia*: *Artemisia frigida*;**

***B. glandulosa: Betula glandulosa*;**

***B. pumpellianus: Bromus pumpellianus*;**

***C. filifolia*: *Carex filifolia*;**

***C. purpurascens: Calamograstis purpurascens*;**

***E. spicatus*: *Elymus spicatus*;**

***E. trachycaulus: Elymus trachycaulus*;**

***F. altaica*: *Festuca altaica*;**

***L. lewissi*: *Linum lewisii*;**

***L. ramosissimum: Lepidium ramosissimum*;**

***P. canescens: Plantago canescens*;**

***P. glauca*: *Poa glauca*;**

***P. gormani: Penstemon gormanii*;**

***R. idaeus*: *Rubus idaeus*;**

***S. arctica*: *Salix arctica*;**

**FR: Fine root; RC: Root crown; S: Stem; L: Leaf; I: Inflorescence.**

**G: Grass; S: Shrub; SS: Subshrub; SG: Sedge; F: Forb.**

**Boldface denotes average of duplicates.**
