# Supplementary figures and images for: Nitrogen and carbon isotopic dynamics of subarctic soils and plants in southern Yukon Territory and its implications for paleoecological and paleodietary studies
Source: PLoS One. 2017 Aug 16;12(8):e0183016. doi: 10.1371/journal.pone.0183016 (PMC5559067; doi:10.1371/journal.pone.0183016)

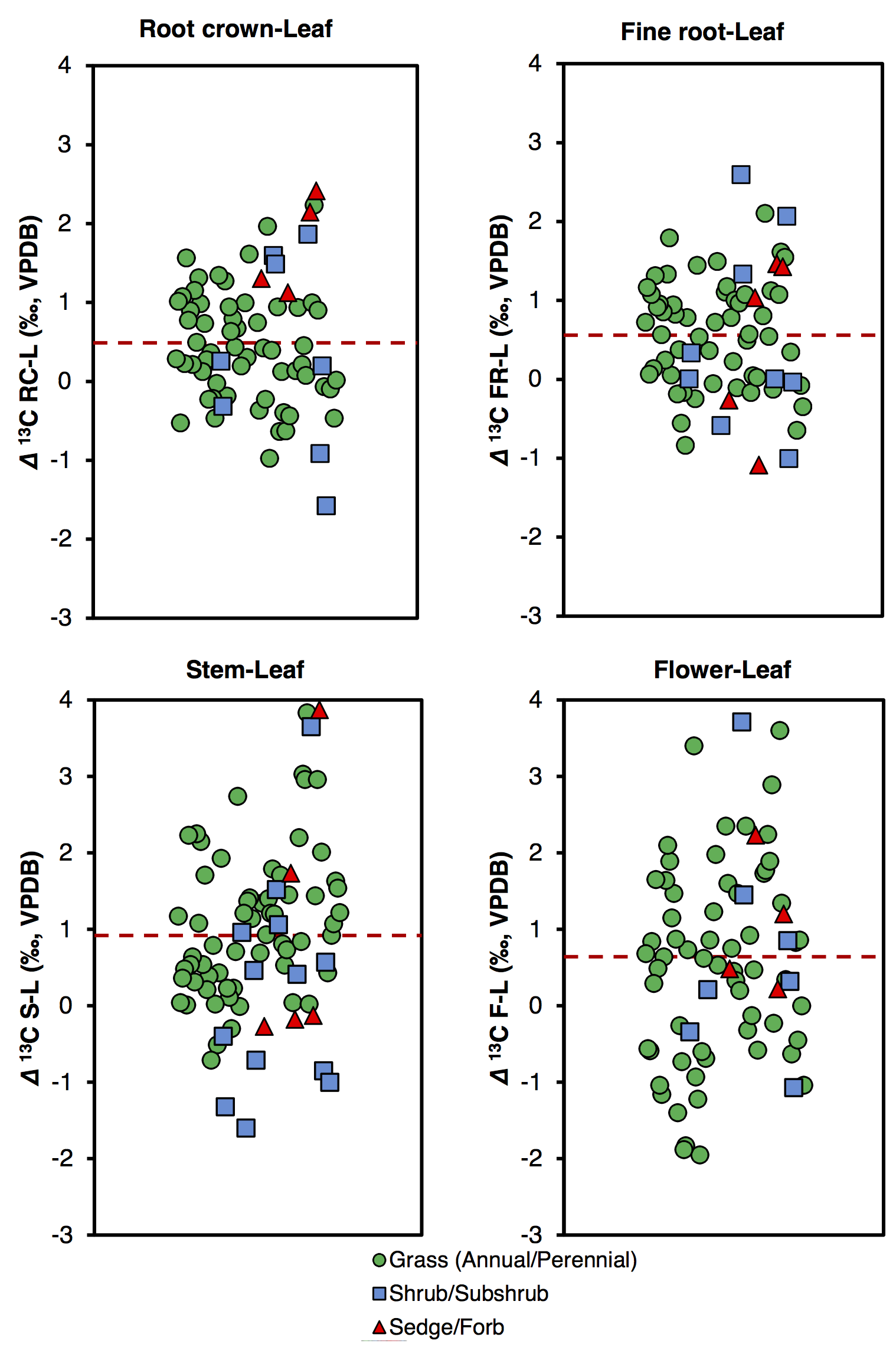

Supplement: S1 Fig — Dashed lines represent means. (TIFF) [file pone.0183016.s004.tiff]

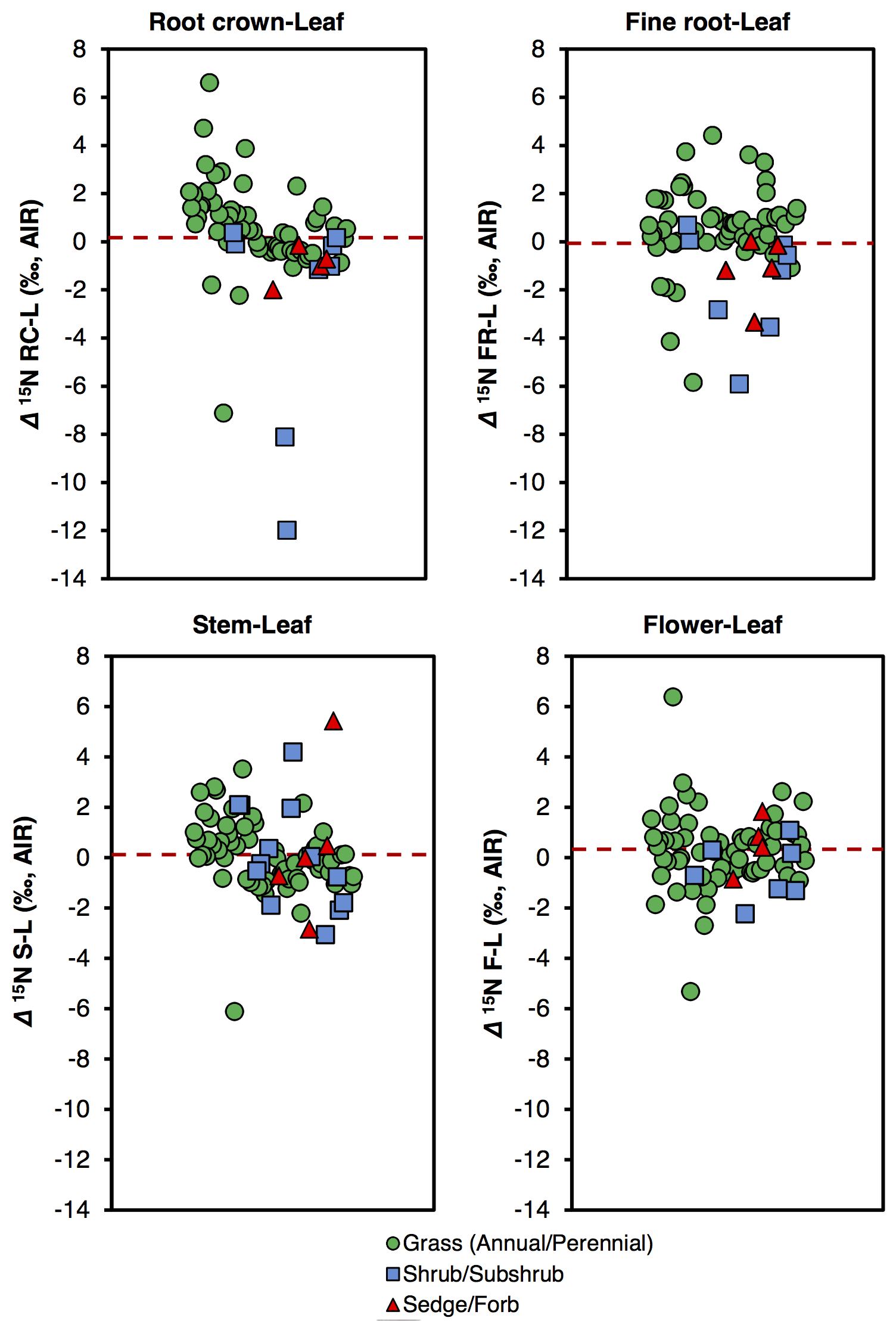

Supplement: S2 Fig — Dashed lines represent means. (TIFF) [file pone.0183016.s005.tiff]
